# Evolutionary maintenance of filovirus-like genes in bat genomes

**DOI:** 10.1186/1471-2148-11-336

**Published:** 2011-11-17

**Authors:** Derek J Taylor, Katharina Dittmar, Matthew J Ballinger, Jeremy A Bruenn

**Affiliations:** 1 Department of Biological Sciences, The State University of New York at Buffalo, Buffalo, NY 14260, USA

## Abstract

**Background:**

Little is known of the biological significance and evolutionary maintenance of integrated non-retroviral RNA virus genes in eukaryotic host genomes. Here, we isolated novel filovirus-like genes from bat genomes and tested for evolutionary maintenance. We also estimated the age of filovirus VP35-like gene integrations and tested the phylogenetic hypotheses that there is a eutherian mammal clade and a marsupial/ebolavirus/Marburgvirus dichotomy for filoviruses.

**Results:**

We detected homologous copies of VP35-like and NP-like gene integrations in both Old World and New World species of *Myotis *(bats). We also detected previously unknown VP35-like genes in rodents that are positionally homologous. Comprehensive phylogenetic estimates for filovirus NP-like and VP35-like loci support two main clades with a marsupial and a rodent grouping within the ebolavirus/Lloviu virus/Marburgvirus clade. The concordance of VP35-like, NP-like and mitochondrial gene trees with the expected species tree supports the notion that the copies we examined are orthologs that predate the global spread and radiation of the genus *Myotis*. Parametric simulations were consistent with selective maintenance for the open reading frame (ORF) of VP35-like genes in *Myotis*. The ORF of the filovirus-like VP35 gene has been maintained in bat genomes for an estimated 13. 4 MY. ORFs were disrupted for the NP-like genes in *Myotis*. Likelihood ratio tests revealed that a model that accommodates positive selection is a significantly better fit to the data than a model that does not allow for positive selection for VP35-like sequences. Moreover, site-by-site analysis of selection using two methods indicated at least 25 sites in the VP35-like alignment are under positive selection in *Myotis*.

**Conclusions:**

Our results indicate that filovirus-like elements have significance beyond genomic imprints of prior infection. That is, there appears to be, or have been, functionally maintained copies of such genes in mammals. "Living fossils" of filoviruses appear to be selectively maintained in a diverse mammalian genus (*Myotis*).

## Background

While genomic transfers from retroviruses to eukaryotic hosts are well known and expected, transfers from non-retroviral RNA viruses to eukaryotes are unexpected [1]. Non-retroviral RNA viruses lack the coding for reverse transcriptase and the integration machinery needed for successful transfer to DNA genomes. However, several recent studies have provided evidence for widespread viral transfer to fungi[2-4], animals [3,5-10], and plants [3]. These transfers have been termed NIRVs (non-retroviral integrated RNA viruses) because the integrated elements differ from endogenous viruses in their requirement for co-option of integration machinery and in their normally subgenic architecture [3,4,10,11]. A NIRV is a subclass of paleovirus or endogenous viral element (EVE,[8]). Unlike other paleoviruses, there is no evidence that a NIRV has ever coded for an active endogenous viral genome or is capable of copying itself as with endogenous retroviruses. Several studies have implicated and identified the signatures of retrotransposon activity in association with NIRV formation [3,4,6,7,10].

Because NIRVs are a form of "fossil" viral element, their recognition permits, for the first time, study of the deeper evolution of non-retroviral RNA viruses and an understanding of the timescale of genomic interactions. The ages of NIRVs have turned out to be much older than those age estimates from molecular clocks based on nucleotide substitution rates. Horie et al. [7], for example, assigned a date of >40 My for the integration of bornaviruses into primate genomes based on phylogenetic clades of NIRVs from genome assemblies. The determination of orthology can be complicated by gene duplications and horizontal transfers of NIRVs among hosts [4]. Homology is unambiguous when the dated NIRVs are monophyletic and share integration locations (i.e., synteny or positional homology). Taylor et al. [10] provided a minimum date of about 10 million years for filovirus-like NP gene NIRVs based on the shared integration location and monophyly in rodents that have dated fossil records. Still, the timescale of host interactions for nonretroviral RNA viruses (including those viruses with NIRVs) remains poorly studied.

The biological significance of NIRVs beyond that of a viral fossil record remains controversial. Have NIRVs been co-opted by eukaryotic hosts for a novel or an antiviral function? Thus far, evolutionary evidence for maintenance of NIRVs among host species has been ambiguous. Although the vast majority of known NIRVs have disrupted open reading frames (ORFs), extended ORFs and RNA transcripts of NIRVs have been identified in mosquitoes [6], yeast [4], primates [5,7] and plants [3]. But these cases of NIRV RNA expression involved only one or two species per group and, with the exception of the *Arabidopsis *IAA-leucine-resistant protein 2 (ILR2;[3,12]), could be the result of transcriptional noise. Evidence of significant selective maintenance among species in the yeast-totivirus NIRV is complicated by the dearth of known orthologs [4]. There is some evidence for evolutionary maintenance in the mammal-filovirus-like NP NIRV system [4,10] and in the primate-Borna virus systems [8]. But in each case, a substantial proportion of the species in the analyses of NIRV maintenance had disrupted ORFs. As such, the existing evolutionary analyses of NIRVs in mammals indicate more a slowed rate of erosion in codon structure than selection for maintenance of an ORF [13]. Presently, it is difficult to determine when or if selective maintenance has occurred in known NIRVs because the comparisons normally involve sparse taxonomic sampling and distantly related host genome assemblies. An improved test of the selective maintenance of NIRVs would involve a comparison of regions that lack ORF disruptions and have demonstrated positional homology within a closely related group of animals such as a genus.

Filovirus-like NIRVs in mammals are candidates for a more detailed study of selective maintenance. Taylor et al. [10] isolated copies of the NP-like gene from specimens of the big brown bat (*Eptesicus fuscus*), the little brown bat (*Myotis lucifugus*) and the tammar wallaby (*Macropus eugenii*), but each of these NIRVs appeared to be pseudogenized. Belyi et al. [5] detected an extended open reading frame of a VP35-like gene from a BLAST query of the NCBI genome assembly of the little brown bat (*Myotis lucifugus*). As filoviral VP35 has been shown to interfere with host defences, the acquisition of a mammalian NIRV has been proposed as a putative co-option to interfere with viral infection [5]. The presence of an open reading frame of a NIRV in a single genome assembly could be associated with function or merely indicate a recent integration where there has been insufficient time to accrue ORF disruptions. Pseudogenization for bats could also be a slow process compared to similar-sized mammals, requiring an estimated 2.02 My of neutral evolution just to reach a 50% probability of ORF disruption [14]. Several mammals including mouse and rat have been identified as possessing the filovirus NP-like NIRVs [10], but only the tarsier, tammar wallaby, and little brown bat have been identified as possessing the VP35-like NIRVs [5]. None of the BLAST-matched VP35 NIRVs have been verified by independent DNA sequencing. One limitation of the BLAST approach for identifying NIRVs is the tendency for underestimation of NIRVs when only known viral genes are used as queries. When clades of NIRVs are detected that are divergent from known viruses, their membership might be underrepresented. One way around this problem is to carry out secondary BLAST searches with the divergent NIRVs as queries [10]. More NIRV sequences from VP35 would be particularly important in testing the hypothesis [10] that known filoviruses form two divergent clades (Marburgviruses, ebolaviruses with marsupial NIRVs) and (placental mammal NIRVs with an unidentified filovirus clade). It is presently unclear if the VP35-like NIRVs form the same phylogenetic associations with known filoviruses [5,15] as found with the other filovirus-like NIRVs.

Here we test for the evolutionary maintenance of filovirus VP35-like and NP-like NIRVs in the bat genus *Myotis. Myotis *is a diverse genus of mammals (> 100 species) and is thought to have radiated from Asia to every continent save Antarctica over the past 13.4 My [16,17]. There are several congruent studies of both nuclear and mitochondrial genes [16-18] that find three main geographic clades of *Myotis *(North America, South America and Old World). We test positional homology by PCR amplifying between the VP35 NIRV and a neighboring gene. We also compare the evolution of NP and VP35-like NIRVs in *Myotis*. The results provide evidence that filovirus-like integrations are more widespread in mammals than previously thought and that these transferred genes have been exposed to positive selection and selection for open reading frames in bats.

## Results and Discussion

tBLASTn searches of the WGS database using the filovirus-like VP35 NIRV of *Myotis lucifugus *(i.e., the ORF of the genome project: AAPE02000262.1, 88641-89459) as a query sequence, yielded three previously unknown mammal species matches with expect values <10^-5 ^(Additional file 1, Table S1). The Chinese hamster (*Cricetulus griseus*), mouse and rat had significant matches using this bat query, with hamster having the best match for these rodents. The strongest match in rats (mapped to chromosome 9) had a highly significant BLAST match to the mouse genome (CAAA01163972.1; 1e-34; Chromosome 1). We note that the known marsupial VP35-like NIRV did not appear as a match when the *Myotis lucifugus *sequence was used as a query, presumably a result of a BLAST analysis where subjects are from divergent clades. When the VP35 of Marburgvirus was used as a query, different contigs of the Chinese hamster (*Cricetulus griseus*) had the best matching sequences in the WGS. Likewise, when NP of Marburgvirus was used as a query, the Chinese hamster again had the best match. We also detected a new NP-like NIRV from the naked mole rat (that is, in addition to those detected by Taylor et al. [10]).

**Figure 1:**
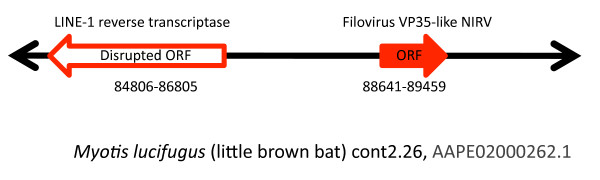
**Map showing the position of the filovirus VP35-like gene and the nearby LINE-1 reverse transcriptase gene in bats of the genus *Myotis***. The solid arrows represent an open reading frame (ORF), and the hollow arrow represents a disrupted ORF. Numbers below the arrow show the boundaries of the annotation in the contig.

We tested for positional homology and integration by DNA sequencing PCR products in *Myotis*. A translated BLAST of the *Myotis lucifugus *contig (AAPE02000262.1) with the VP35-like NIRV revealed a neighboring LINE-1 reverse transcriptase element (Figure 1) with the best match being from *Bos Taurus *(AAY53484.1; E value = 1e-110; Maximum identity = 71%). A nucleotide BLAST using the primers from the LINE-1 element and the VP35-like region as queries, and the *Myotis lucifugus *genome assembly (WGS) as a database, yielded only a single match to the targeted region of cont2.261 (AAPE02000262). DNA sequence from a PCR product using a primer in this LINE-1 element and a primer in the VP35-like region suggested the positional homology the *Myotis *sp. sequences used in the present study. Two of the PCR products (*M. albescens *and *M. horsfieldii*) for the intergenic region failed to yield clean sequence so an additional intergenic primer was used. Another PCR was carried out to obtain overlapping sequence from the remaining VP35-like region in the examined bat specimens. Although all of the bats examined in the genus *Myotis *had a VP35-like element, none of the other genera of bats assayed for VP35 yielded a positive amplicon (including other vesper bats). NP-like amplicons were obtained in several species of bat, but reliable sequence was obtained from only *Myotis *and *Eptesicus*. More genome assemblies of bats are needed to determine if this lack of a match is due to sequence divergence of the primer sites or to the lack of filovirus-like NIRVs. Still, the finding of VP35-like DNA sequence with the same upstream neighboring LINE-1 element in the genus *Myotis *establishes positional homology for the VP35-like elements. We also note that an Old World species (*Myotis blythii*) has the same putative 5' direct repeat and canonical transcription start site (of LINE-1 elements) in the same location (+3 bp from indels) as that identified by Belyi et al. [5] for the New World species, *Myotis lucifugus*. Strongly repetitive sequence appears to have shortened our sequence reads for other species of bats in the intergenic area. Our result supports homology among bat inserts for VP35 and the hypothesis that bat NIRVs result from target-primed reverse transcription of filoviral mRNA by LINE elements [5,10]. The positional homology of the previously unknown rodent VP35-like sequences was established by comparisons of synteny between mouse and rat genomes. The VP35-like element on CHR 9 of the rat and on CHR 1 of the mouse appear to be in markedly syntenous genome locations, supporting positional homology (Additional file 2, Figure S1).

**Figure 2:**
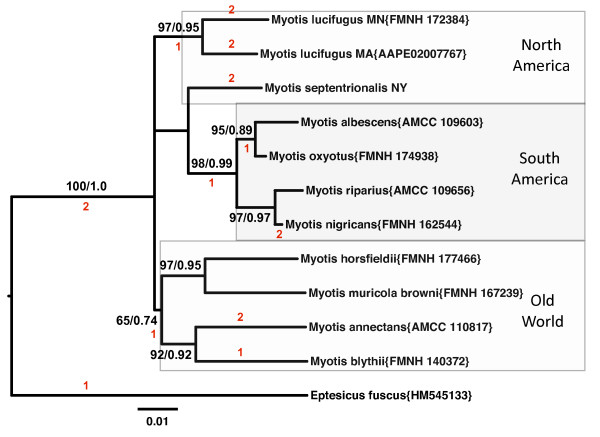
**Midpoint rooted maximum likelihood phylogram of filovirus-like nucleoprotein (NP) nucleotide sequences from vespertilionid bats**. Three major geographic clades of the genus *Myotis *are shown by shaded boxes -- the big brown bat (*Eptesicus fuscus*) sequence has been added for comparison and to show the rooting that divides Old and New World *Myotis*. Support values (nonparametric bootstrap support/approximate likelihood ratio tests) are given above the branches. GenBank accession numbers or museum voucher numbers are provided in parentheses. See Additional file 8, Table S3 for details of specimen records and acronyms. Red numbers indicate the sum and presumed phylogenetic position of independent observed disruptions to the open reading frame. The scale bar is based on substitutions per site.

**Figure 3:**
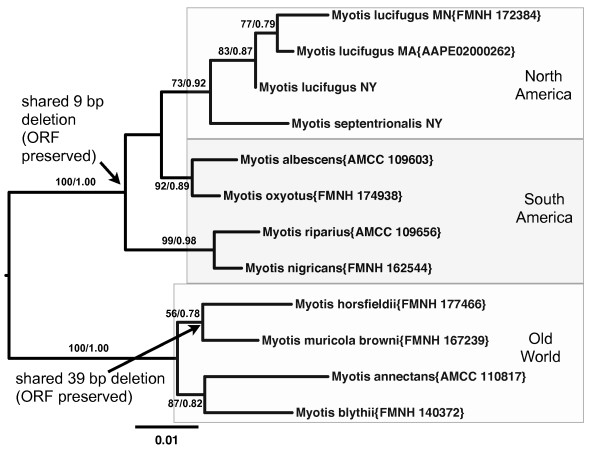
**Midpoint rooted maximum likelihood phylogram of filovirus-like VP35 nucleotide sequences from vespertilionid bats**. Three major geographic regions are shown by the shaded boxes. Support values (nonparametric bootstrap support/approximate likelihood ratio tests) are given above the branches. GenBank accession numbers or museum voucher numbers are provided in parentheses. See Additional file 8, Table S3 for details of specimen records and acronyms. Arrows show unique indels shared by sister taxa that preserve the open reading frame (i.e. all sequences appear to have an open reading frame in the genus *Myotis*). Note that the 9 bp deletion is not observed in the homologous sequence from the tarsier (supporting its derived position in New World *Myotis *sp.). The scale bar is based on substitutions per site.

More evidence of homology for the filovirus-like sequences in the genus *Myotis *is provided by phylogenies that match the expected species trees. Prottest determined that the JTT+G model was the best fit with the AIC criterion for both amino acid alignments. The K80+G model was determined to be the best model for the nucleotide alignments of the two nuclear genes. The TN93+G+I substitution model was determined to be the best-fit model for the mitochondrial gene sequences of the genus *Myotis*. For each of the three genes (mitochondrial cytochrome oxidase subunit I, VP35-like NIRVs, NP-like NIRVs) sequenced, the estimated phylogenetic tree was similar in topology. That is, there is a North American, South American, and Old World clade for most genes (Figures 2,3; Additional file 3, Figure S2). This finding suggests that for each NIRV, the integration predated the radiation of *Myotis *from Asia. The relationships within each continent are also nearly identical across genes. For example, the Neotropical species *Myotis riparius *and *M. nigricans *are strongly supported as sister taxa in every gene tree. Most of the specimens were closely related to conspecifics on the mitochondrial gene tree (Additional file 3, Figure S2A). Sequences of *Myotis blythii *and *Myotis muricola browni *were distantly related to known mitochondrial sequences from the genus *Myotis *or others from the present study. Consistent with our results, Stadelmann et al. [17] concluded that specimens identified as *M. muricola browni *from the Phillipines belonged to an undescribed or different species from *M. muricola*. We also note that the specimen identified as *M. nigricans *groups with one of the two known clades of *M. riparius *on the COI tree and not with the *M. nigricans *clade. This discordance with species names could be a reflection of the difficulty in morphological diagnosis of species in the genus. We detected three copies of NP-like genes and one copy of the VP35-like gene in the most recent genome assembly for *Myotis lucifugus *(Myoluc 2.0; released September 2010). The two additional NP-like copies in *M. lucifugus *form a distinct basal clade to the NP-like gene sequences isolated in the present study from bats (see the amino acid-based phylogeny in Additional file 3, Figure S2B). However, the two basal paralogs lack significant similarity at the nucleotide level (as indicated by a BLAST search) to the NP-like sequences from the present study. Where there are additional disagreements among genes, the support values are weak, suggesting a lack of signal rather than non-orthology. Similar topological patterns are observed for the VP35 and NP phylogenies in bats based on amino acid alignments (Additional file 3, Figures S2B, C). The largely congruent topologies for each gene to the expected species tree supports the notion that the NIRV formations occurred before the *Myotis *species radiation and geographic expansion to the New World. That is, the NIRV copies that we isolated appear to be ancient orthologs.

The determination of orthology and positional homology permits estimation of minimum ages of viral-host associations. For *Myotis*, the VP35-like gene is estimated to have had an ORF for at least 13.4 My (11-18 My with error bars). The timescale estimates are based on the dating of the divergence of *Myotis *with multigenic molecular analyses and at least two internal fossil calibrations [16,17]. The insertion of the NP NIRV predates the common ancestor of *Eptesicus *and *Myotis*, which has been estimated at 25 My (19-30 My range) using multiple fossil calibrations and loci [19]. The proposed minimum age of the *Rattus*/*Mus *common ancestor based on dating of the oldest fossil record of the genus *Progonomys *(the presumed genus of the *Rattus*/*Mus *common ancestor) is 12.3 My [20]. Although knowledge of the timescale of mammalian radiations is in a state of flux, it is clear that NP-like and VP35-like genes are ancient and independently integrated in rodents and in bats.

**Figure 4:**
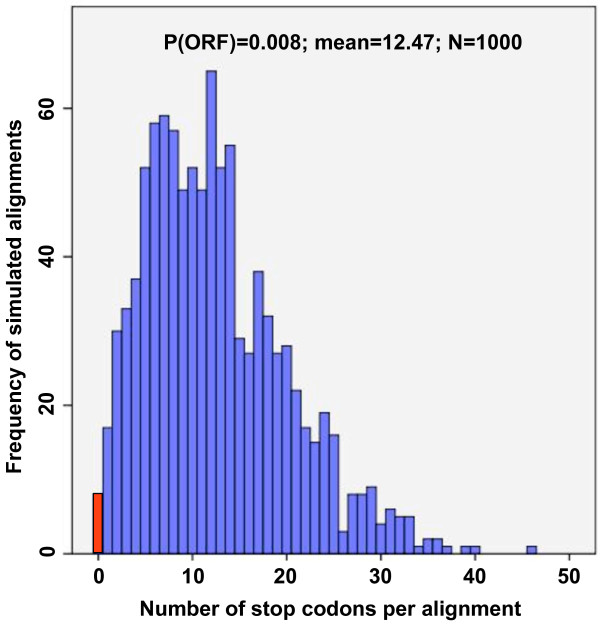
**Histogram showing the distribution of expected disruptions in open reading frame in 1000 simulations of nucleotide evolution of filovirus VP35-like sequences in the bat genus *Myotis***. The simulations start from a center of tree (COT) ancestor with an ORF and use the best-fit model of substitution and the observed maximum likelihood tree with branch lengths as parameters. The observed number of disruptions (zero) is shown by a red bar - a value found in only 0.008 of the simulations. Note that the simulations are conservative because they do not account for selective maintenance involving indel mutations (see Figure 3).

The two NIRV loci in *Myotis *appear to be evolving under different selective regimes. The ORF for VP35-like elements has been maintained during the radiation (Additional file 4, Figure S3). The few heterozygous sites found also failed to disrupt the reading frames. Further analyses indicate that the lack of observed ORF disruptions in the genus is unlikely to be due to chance or to a lack of sufficient evolutionary time for disruption. First, independent deletions (9 bp and 39 bp) are observed that fail to disrupt the reading frame (Additional file 4, Figure S3). As about 83% of indel mutations are expected to disrupt the ORF [21], it is unlikely (P = 0.0289) that two independent large indels would be shared by several taxa and still maintain the open reading frame over millions of years of neutral evolution. As most indel mutations are less than eight nucleotides [22], the presence of only large indels in the VP35-like region of *Myotis *suggests that many smaller indels have been culled by selection. But, even if indels are ignored as a source of ORF disruptions, our simulations indicate that at least twelve premature stop codons are expected per alignment under neutral evolution for the VP35-like element in *Myotis *(Figure 4). The K80+G model was input for the parametric simulation (where kappa = 2.48 and gamma shape = 0.234). The observed value of zero stop codons per alignment had a frequency of 0.007 in the parametric simulations under no selection for ORF maintenance. Thus, with over 13 My of neutral evolution in *Myotis*, ORFs in the VP35-like region are not expected to be observed from chance alone. The same timescale of evolution revealed ongoing ORF disruption of the NP-like NIRV (with a similar alignment length and genetic divergence as the VP35 region) elements of bats (Additional file 5, Figure S4). At least one ORF disruption of the NP-like NIRVs is shared among species of *Myotis*. Taken together, the indel and simulation analyses suggest that there has been selective maintenance of the ORF for the VP35-like genes but not for the NP-like genes in *Myotis *during their radiation across continents. Finally, Bayesian evolutionary models that accommodate codon usage differences revealed a pattern of positive selection for the VP35-like genes. A model that allows for positive selection (Model M8; Likelihood = -2121.07) was a better fit (according to a Likelihood ratio test that had a significance level of P = 0.001) than a model that does not allow for positive selection (Model M8a; Likelihood = -2126.93). Eliminating the *lucifugus *sequence from NY to yield the same taxon set as the NP gene alignment still resulted in a significance level of P = 0.01. Site specific analysis in Selecton indicated that 32 codon positions in the VP35 NIRV alignment had lower confidence interval bounds for K_a _/K_s _of > 1, indicating positive selection at these sites (Additional file 6,Table S2). Similarly REL analysis in HyPhy found 25 sites (each also found by Selecton) to be under significant positive selection (Additional file 6,Table S2). The NP-like gene alignment for *Myotis *lacked a significant improvement in fit with a model that accommodated positive selection (Model M8; Likelihood = -2031.48; Model M8a; Likelihood = -2033.26; P = NS). Our results suggest that filovirus-like elements have been co-opted by bats and that a VP35-like gene has been well maintained and modified by selection for over > 13 My.

**Figure 5:**
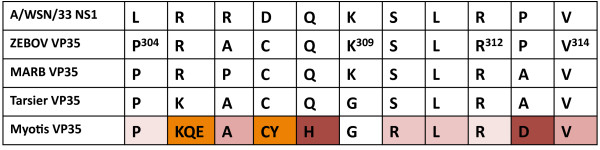
**Comparison of the presumptive residues of the interferon-binding region from filoviruses and filovirus VP35-like regions**. Bat residues are colored according to a site-by-site K_a _/K_s _analysis according to the output from Selecton (i.e., dark purple indicates strong purifying selection and dark orange indicates strong positive selection). See Additional file 6,Table S2 for statistical details and values of K_a _/K_s _for the full alignment.

We attempted to carry out RT-PCR of the VP35-like region in *Myotis lucifugus *to test for gene expression. However, no RNA product was detected, despite observing an RNA band for the actin gene (Additional file 7, Figure S5). It remains unknown if there is an expression product for VP35 in bats or if expression is induced under specific conditions such as filoviral infection. Although filovirus NP-like genes have previously been detected in marsupial EST databases [10], we detected no VP35-like NIRVs in EST databases. It is clear that filoviruses have undergone significant coevolution with mammalian hosts as they have developed several mechanisms to counter mammalian host immune responses. Notably, some of these countermeasures involve VP35-mediated suppression of RNA silencing [23], dsRNA binding activity and antagonism of the interferon response[24]. As Belyi et al. [5] note, VP35-like gene expression could be a coevolutionary answer of mammals to the multifarious immunosuppressive role of viral VP35. While at least two sites in the interferon-binding motif of VP35 of *Myotis *appear to have undergone selection to change their residues, most other residues in this area appear to be exposed to varying degrees of purifying selection (Figure 5). Although there has been significant progress in understanding the function and structure of VP35 in ebolaviruses, it is important to realize that functions such as interferon inhibition likely differ among filoviruses [25] -- the clade that contains the bat VP35-like genes is quite divergent from ebolaviruses. In essence, the functional interactions of the filovirus-VP35-like NIRVs with viral products remain unknown. Ideally, closely related exogenous and endogenous filoviruses will be discovered that will enable the development of an experimental system to study function.

**Figure 6:**
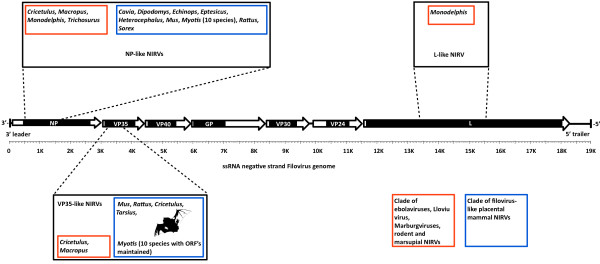
**Cartoon of idealized genome map of a filovirus showing known mammalian NIRVs and their two major clades**. Shown are three gene regions of filovirus genomes that are known to have mammalian NIRVs. Red and blue colored boxes indicate phylogenetic clades. The bat cartoon indicates the gene region in *Myotis *that appears to have an open reading frame maintained.

Our results show that VP35-like and NP-like NIRVs are even more taxonomically widespread in mammals than thought (Figure 6). With our discovery of new NIRVs in rodents and 10 species of vespertilionid bats, the results support two major clades of filovirus-like NIRVs in mammals. However, these clades lack a strict association with placental and marsupial mammals (indeed the hamster has NIRVs in both clades). One clade contains ebolaviruses, Lloviu virus, Marburgviruses, marsupials and the Chinese hamster, and the other contains only placental mammals (Additional file 3, Figures S2B, C). The hamster and the wallaby genomes contain NIRVs within the ebolavirus/Marburgvirus clade. Note that Taylor et al. [9] found the same root location that we found here when they used related (non-filoviral Mononegavirales) sequences for outgroup rooting (NP and L genes). The filovirus-NIRV phylogenies are consistent with host jumps between placental and marsupial mammals. However, more NIRVs from mammalian genomes are needed to address the timing of host switches. The recently discovered Lloviu virus [26,27] from a bat (*Miniopterus schreibersii*), is only distantly related to the NIRVs from bats (*Myotis*) in the present study. However, closely related NIRVs and exogenous viruses seem to be rare -- a pattern predicted by the antiviral hypothesis for NIRVs. Viromes of New World vespertilionid bats, including filovirus NIRV-containing *Myotis lucifugus *and *Eptesicus fuscus*, have been shown to be very diverse, but lacking in filoviruses [28,29]. Experimentation is needed to directly test the antiviral function hypothesis for NIRVs in bats.

## Conclusions

The results indicate that the largest species radiation in mammals could be associated with the maintenance of a "living" fossil copy of the VP35-like gene co-opted from filoviruses. The results reveal a rare example of non-retroviral viral genes that have been successfully co-opted by mammals.

## Methods

### Nucleic Acid Extractions

Total nucleic acids were extracted from wing punches (Rabies Laboratory, New York State Health Dept.), or from preserved tissue using the Qiagen blood and tissues nucleic acids extraction kit (Qiagen) or the DNA Quickextract solution (Epicentre^®^). Thus, no live bats were harmed by the extraction of nucleic acids during this study. Sample information with voucher numbers from the Field Museum of Natural History and the American Museum of Natural History are provided in Additional file 8, Table S3.

### PCR, RTPCR, and DNA Sequencing

50 μl PCR reactions were assembled according to the protocol for Takara Primestar HS DNA polymerase with 5 μL of extracted DNA template. Primers for sequencing and PCR were: LINE1 to VP35-like intergenic region (5'-GCCTCCTAAAATGAGTTTGTGAGTGTTCCCTGGTC-3'; 5'-GAGTGGATGTTGCAGGTCCTGACATTACAGGC-3' with an amplicon size of 2365 bp in *Myotis lucifugus*); VP35-like region (5- CTTCTGTCTACGTCTTCTAAGGTTAATC -3; 5- CCCGAGGCTTCCTTCAGGAGTTAG -3; with an amplicon size of 660 bp in *Myotis lucifugus*). A third primer combination was used to fill in gaps in sequences (5'- CTCGTCAGATCAGCATGTCCCTGGAGC -3' and 5'-GAGTGGATGTTGCAGGTCCTGACATTACAGGC-3'). We used the primers of Taylor et al. [10] for the NP-like region and the universal primers of Folmer et al. [30] for the mitochondrial gene. For the NP region, the new genome assembly appears to have introduced a deletion (AAPE02007767) for *Myotis lucifugus*. We used the latest assembly in the present paper. The PCR temperature profiles were: 10 cycles of 94°C for 30 s, 59°C for 30 s and 72°C for 2 min, with a touchdown to an annealing temperature of 48°C over 30 additional cycles and a final extension at 72°C for 5 min. A constant annealing temperature of 45°C was used for the mitochondrial primers. PCR products were purified and sequenced by the University of Washington High Throughput Genomics Facility. Geneious 4.8 was used to assemble and edit electrophoregrams. For RT-PCR, total nucleic acids were extracted from a frozen specimen of *Myotis lucifugus*. The internal organs were ground in liquid nitrogen and a subsample was exposed to extraction. RNA templates were exposed to DNAse. The Qiagen One step RT-PCR kit was used with VP35-like primers and primers for the actin gene (5'-ACAGGTCCTTACGGATGTCG-3'; 5'-TATACGCTTCTGGCCGTACC-3') specific to *Myotis lucifugus*. New sequences from this study have the following Genbank accession numbers: JN847695-JN847723.

### Bioinformatics

We searched for sequence similarity to filoviruses using protein sequences based on the VP35 and the NP regions of *Marburgvirus *(NC_001608.3) as a query with tBLASTn in the WGS database of NCBI. Additional searches (tBLASTn) in each of the available NCBI databases used the sequence of *Myotis lucifugus *as a query. Nonviral subject sequences with expect values of E<10^-5 ^and matches greater than 100 amino acid residues were retained for phylogenetic analyses. VP35 sequences from available filoviruses that differed at the AA level were also added to the alignment. Taxonomy of filoviruses followed Kuhn et al. [26]. Filovirus-like Bat sequences from Taylor et al.[10] were added to the NP-like analysis from the present study. Mitochondrial sequences available at NCBI for the genus *Myotis *were retained for the COI gene alignment.

For genome assembly sequences, the sequence boundaries and translations identified by tBLASTn were used to retrieve nucleotide sequences and assemble amino acid sequences. MAFFT [31] was used to align the protein sequences for the VP35 and NP analyses using the JTT100 model. Other alignments were unambiguous, requiring no or few indels.

Phylogenetic estimates were obtained with a maximum likelihood optimality criterion in PhyML 3.0 [32]. Models were chosen according to the best available optimal model from Modeltest [33] or Prottest [34] (ML). Reliability was assessed by approximate likelihood ratio tests (aLRT: SH like tests) and/or posterior probabilities. For PhyML, SPR search algorithms were used with five random starting trees.

Tests of selection were carried out using both gene-wide Bayesian methods [35] and site-specific tests_. _Gene-wide significance was assessed by comparing the fit of a codon-based substitution model that permits sites with positive selection (M8;[36]) to the fit of a null model that does not allow for positive selection (M8a; [37]). This comparison was carried out with likelihood ratio tests (LRTs) where DF = 1 [36]. The test statistic for the LRT is calculated as twice the difference between the likelihood scores of the null model and the alternative model. A Chi- square table is used to obtain the significance. Unlike standard methods based on overall estimates of K_a _/K_s_, codon-based models can account for among-site variation in K_a _/K_s _by assigning sites to discrete rate categories. Significant selection at individual sites in the alignment was assessed by two methods: by confidence intervals around the K_a _/K_s _estimates in Selecton and by the REL method [38] implemented in HyPhy [39,40]. Sites with a CI lower bound of K_a _/K_s _that exceeds 1 in Selecton were considered to be under positive selection. Sites with a Bayes factor of > 50 were considered as reliably under selection in HyPhy. As Selecton requires continuous ORFs, disrupted codons were replaced with gaps. For the Bayesian estimate of K_a _/K_s_, an ML tree was input after estimation with PhyML. Selection for ORF maintenance was estimated using a parametric simulation approach modified from Katzourakis and Gifford [8]. A centre of tree (COT) sequence was estimated using DIVA [41,42]. The COT sequence, which had an open reading frame, was used as a starting sequence for simulated neutral evolution with substitution and branch length parameters input from the observed data. Seq-gen was used to carry out 1000 evolutionary simulations from the COT sequence [43]. The simulated alignments were translated and visualized in Geneious and the number of ORF disruptions per alignment was tallied. A histogram of the ORF disruptions per simulated alignment was created in PASW statistics 18. The probability that an alignment would have complete ORFs by chance was determined from the frequency of alignments with complete ORFs in the parametric simulation.

The orthology of filovirus-like VP35 genes in rat and mouse was assessed by genomic BLAST searches and visualized on the NCBI chromosome maps. We used the Cinteny server [44] and Roundup database [45] for whole chromosome comparisons of larger orthologous blocks.

## Authors' contributions

DJT, KD, MJB and JB conceived the study, carried out the bioinformatics analysis, participated in lab experiments and co-wrote the paper. All authors read and approved the final manuscript.

## Supplementary Material

Additional file 1 **Table S1. tblastn results in subjects of the Whole Genome Shotgun Sequences (WGS) database using three filovirus-like queries**. tblastn results (sequences producing significant alignments) using A. the filovirus-like VP35 element of *Myotis lucifugus *as a query, B. the filovirus-like VP35 element of Marburgvirus NC_001608.3 as a query, and C. the filovirus-like NP element of Marburgvirus NC_001608.3 as a query Click here for file

Additional file 2 **Figure S1. Chromosome maps showing synteny of regions flanking the filovirus-like VP35 elements in rat and mouse genomes**. A. whole chromosome view showing the five synteny blocks found between CHR 1 of the mouse and CHR 9 of the rat and B. local view showing the pronounced positional homology of the filovirus-like elements and flanking genes. Click here for file

Additional file 3 **Figure S2. Midpoint rooted maximum likelihood phylograms with support values**. A. mitochondrial Cytochrome Oxidase I gene for bats of the genus *Myotis*, B. NP gene amino acid sequences from filoviruses and related mammalian genomic elements, and C. VP35-like gene amino acid sequences from filoviruses and related mammalian genomic elements. Note that hamster and wallaby sequences are positioned within the known modern filoviruses. Click here for file

Additional file 4 **Figure S3. Alignment of filovirus VP35-like nucleotide sequences isolated from bat genomes showing open reading frames**. A graphical alignment of the VP35-like region in *Myotis *with open reading frames followed by a FASTA formatted alignment. Note that the two large indels fail to disrupt the open reading frames. Click here for file

Additional file 5 **Figure S4. Alignment of filovirus nucleoprotein (NP)-like nucleotide sequences isolated from bat genomes**. A graphical alignment of the NP-like region in *Myotis *followed by a FASTA formatted alignment. Click here for file

Additional file 6 **Table S2. Site-by-site selection results and statistics for the open reading frame alignment of VP35-like genes in *Myotis *using two methods**. Results of different site-specific tests of positive selection (Bayesian Ka/Ks with model M8; REL analysis in HyPhy) for the filovirus VP35-like gene in *Myotis *bats. Click here for file

Additional file 7 **Figure S5. RT-PCR of VP35-like region from *Myotis lucifugus***. RT-PCR of the filovirus VP35-like region and actin controls in *Myotis lucifugus *from two replicates each from two tissues. Click here for file

Additional file 8 **Table S3. Table showing the specimen details for bats assayed for filovirus-like sequences**. Origin of specimens used for the attempted isolation of filovirus-like sequences in the present study. Click here for file
