# Supplementary material for: Evolutionary maintenance of filovirus-like genes in bat genomes
Source: BMC Evol Biol. 2011 Nov 17;11:336. doi: 10.1186/1471-2148-11-336 (PMC3229293; doi:10.1186/1471-2148-11-336)
Supplement: Additional file 1 — Table S1. tblastn results in subjects of the Whole Genome Shotgun Sequences (WGS) database using three filovirus-like queries. tblastn results (sequences producing significant alignments) using A. the filovirus-like VP35 element of Myotis lucifugus as a query, B. the filovirus-like VP35 element of Marburgvirus NC_001608.3 as a query, and C. the filovirus-like NP element of Marburgvirus NC_001608.3 as a query [file 1471-2148-11-336-S1.PDF]

Table S1A. TBLASTN results (sequences producing significant alignments) using the filovirus-like VP35 element of *Myotis lucifugus* in AAPE02000262.1 as a query and the Whole Genome Shotgun Sequences (WGS) database.

Accession	Species	Total Score	Expect
AAPE02000262.1	Myotis lucifugus cont2.261	560	2e-180
ABRT010013008.1	Tarsius syrichta cont1.13007	185	3e-37
AFTD01116912.1	Cricetulus griseus scaffold3533_7	127	4e-14
AFTD01041572.1	Cricetulus griseus scaffold767_33	100	9e-12
AFTD01048309.1	Cricetulus griseus scaffold731_43	99.4	1e-11
AFTD01042578.1	Cricetulus griseus scaffold803_78	114	3e-10
AABR05163314.1	Rattus norvegicus strain BN/SsNHsdMCW	64.7	8e-08
AAHX01060906.1	Rattus norvegicus strain BN chromosome 9 C	64.7	8e-08
AABR05122655.1	Rattus norvegicus strain BN/SsNHsdMCW	62.0	7e-07
AAHX01035392.1	Rattus norvegicus strain BN chromosome 5 C	62.0	8e-07
AEKR01092846.1	Mus musculus strain C57BL/6J cont1.92845	55.8	6e-05
AEKQ01179158.1	Mus musculus strain C57BL/6J cont1.179157	55.8	6e-05
AAHY01034122.1	Mus musculus strain 129X1/SvJ chromosome 4	55.8	6e-05
CAAA01177961.1	Mus musculus whole genome shotgun assembly	55.8	6e-05

Table S1B. TBLASTN results (sequences producing significant alignments) using the filovirus-like VP35 element of Marburgvirus NC_001608.3 as a query and the Whole Genome Shotgun Sequences (WGS) database.

Accession	Species	Total Score	Expect
AFTD01097896.1	Cricetulus griseus scaffold2396_25	98.6	1e-18
ABQO010378232.1	Macropus eugenii Contig378249	54.7	9e-14
AFTD01014524.1	Cricetulus griseus scaffold262_54	81.3	7e-13
AAPE02000262.1	Myotis lucifugus cont2.261	79.0	4e-12
ABRT010013008.1	Tarsius syrichta cont1.13007	56.6	4e-12
AFTD01108184.1	Cricetulus griseus scaffold2943_32	46.6	5e-08
AFTD01120167.1	Cricetulus griseus scaffold3616_61	56.2	9e-05

Table S1C. TBLASTN results (sequences producing significant alignments) using the filovirus-like NP element of Marburgvirus NC_001608.3 as a query and the Whole Genome Shotgun Sequences (WGS) database.

Accession	Species	Total Score	Expect
AFTD01077804.1	Cricetulus griseus scaffold1684_1	111	1e-53
AFTD01097896.1	Cricetulus griseus scaffold2396_25	139	1e-53
ABQO010804673.1	Macropus eugenii Contig804706	113	2e-49
ABQO010766573.1	Macropus eugenii Contig766603	126	2e-38
AAPE02051704.1	Myotis lucifugus cont2.51703	135	3e-37
ABQO010277157.1	Macropus eugenii Contig277168	119	6e-36
ABQO010381849.1	Macropus eugenii Contig381866	154	5e-35
AAPE02014310.1	Myotis lucifugus cont2.14309	116	6e-30
AAFR03026268.1	Monodelphis domestica cont3.026267	85.9	9e-29
AAPE02007767.1	Myotis lucifugus cont2.7766	95.5	1e-25
AFTD01106947.1	Cricetulus griseus scaffold2981_12	105	1e-22
ABQO010278122.1	Macropus eugenii Contig278133	100	8e-19
ABQO010048551.1	Macropus eugenii Contig48552	84.3	2e-17
ABQO010309592.1	Macropus eugenii Contig309604	54.7	7e-15

AFSB01165578.1	Heterocephalus glaber contig165578	90.9	1e-14
ABQO010853818.1	Macropus eugenii Contig853853	74.7	3e-14
ABQO011152526.1	Macropus eugenii Contig1152601	82.0	8e-13
ABQO010223025.1	Macropus eugenii Contig223034	82.0	4e-12
ABQO010478145.1	Macropus eugenii Contig478165	75.1	7e-10
ABQO010047515.1	Macropus eugenii Contig47516	70.9	1e-08
AAKN02025587.1	Cavia porcellus strain inbred line	65.9	5e-07
ABRO01205910.1	Dipodomys ordii cont1.205909	62.8	1e-06
AALT01167855.1	Sorex araneus cont1.167854	61.6	4e-05
AFSB01003667.1	Heterocephalus glaber contig3667	49.3	5e-05
