# Supplementary material for: Evolutionary maintenance of filovirus-like genes in bat genomes
Source: BMC Evol Biol. 2011 Nov 17;11:336. doi: 10.1186/1471-2148-11-336 (PMC3229293; doi:10.1186/1471-2148-11-336)
Supplement: Additional file 2 — Figure S1. Chromosome maps showing synteny of regions flanking the filovirus-like VP35 elements in rat and mouse genomes. A. whole chromosome view showing the five synteny blocks found between CHR 1 of the mouse and CHR 9 of the rat and B. local view showing the pronounced positional homology of the filovirus-like elements and flanking genes. [file 1471-2148-11-336-S2.PDF]

Fig. S1. Chromosome maps showing synteny of regions flanking filovirus-like elements in rat and mouse genomes with a whole chromosome view (A) and a local view (B). White asterisks represent the locations of the phylogenetic sister copies of filovirus-like VP35 elements. Five synteny blocks were found between CHR 1 of the mouse and CHR 9 of the rat. A close up view (B) shows the pronounced positional homology of the Filovirus-like element and the flanking genes (matching colors). Numbers show the boundaries of the large syntenous block on the respective chromosome maps of mouse and rat.

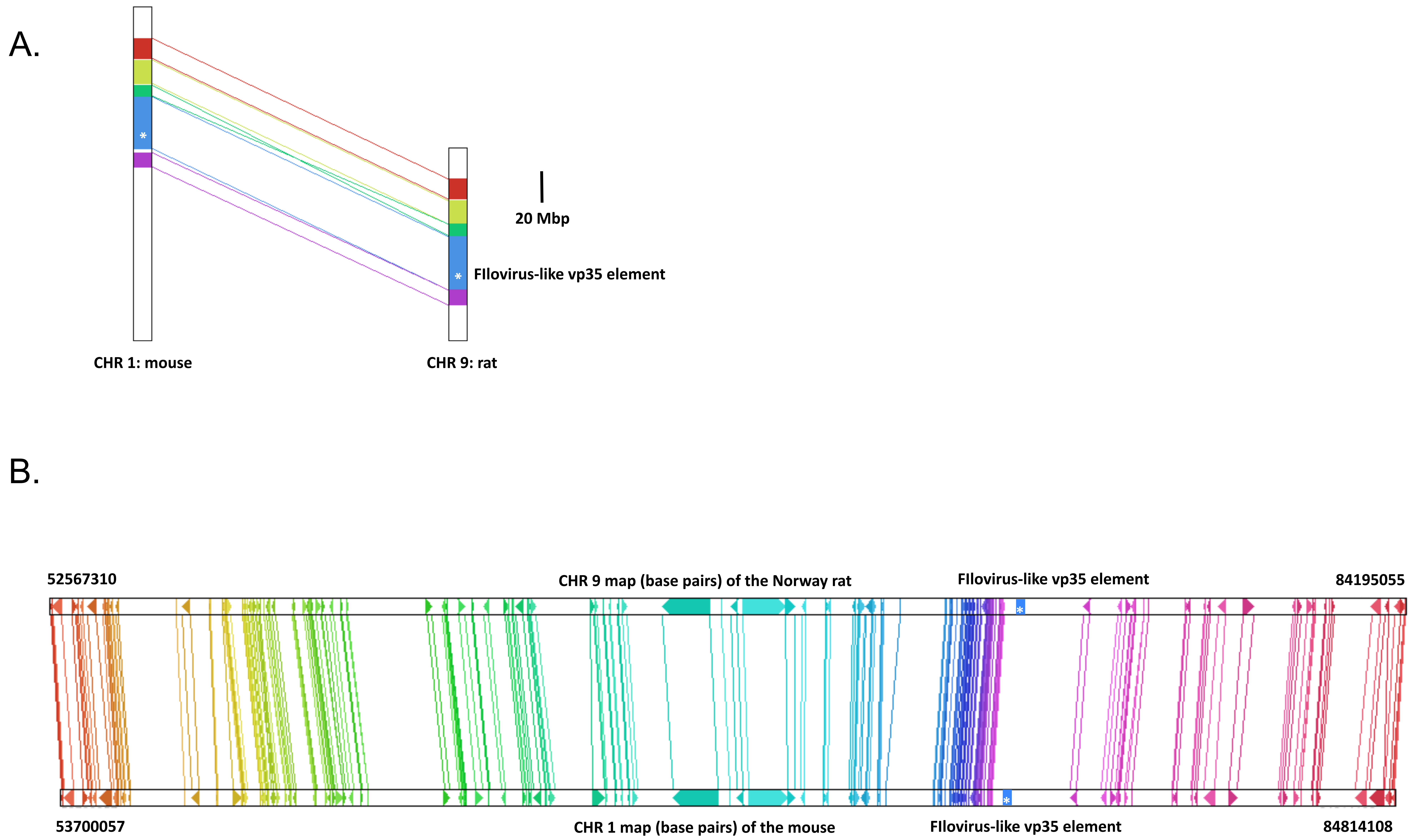

A.
CHR 1: mouse
CHR 9: rat
20 Mbp
Fllovirus-like vp35 element
B.
52567310
CHR 9 map (base pairs) of the Norway rat
Fllovirus-like vp35 element
84195055
53700057
CHR 1 map (base pairs) of the mouse
Fllovirus-like vp35 element
84814108
