# Supplementary material for: Evolutionary maintenance of filovirus-like genes in bat genomes
Source: BMC Evol Biol. 2011 Nov 17;11:336. doi: 10.1186/1471-2148-11-336 (PMC3229293; doi:10.1186/1471-2148-11-336)
Supplement: Additional file 3 — Figure S2. Midpoint rooted maximum likelihood phylograms with support values. A. mitochondrial Cytochrome Oxidase I gene for bats of the genus Myotis, B. NP gene amino acid sequences from filoviruses and related mammalian genomic elements, and C. VP35-like gene amino acid sequences from filoviruses and related mammalian genomic elements. Note that hamster and wallaby sequences are positioned within the known modern filoviruses. [file 1471-2148-11-336-S3.PDF]

Fig. S2A. Midpoint rooted maximum likelihood phylogram based on the mitochondrial Cytochrome Oxidase I gene for bats of the genus *Myotis*. Major geographic clades are shown by shaded rectangles. Support values (approximate likelihood ratio tests) are given on the branches. Sequences from the present study are shaded in red. GenBank Accession numbers or museum voucher numbers are provided in parentheses. B. Midpoint rooted maximum likelihood phylogram of the NP gene amino acid sequences from filoviruses and related mammalian genomic elements. Numbers on branches represent support values (approximate likelihood ratio tests). Parentheses contain GenBank Accession numbers or museum voucher numbers. Red filled branches indicate placental mammal sequences, blue filled branches indicate marsupial mammal sequences and black filled lines indicate viral sequences. Shaded boxes show the two major clades. C. Midpoint rooted maximum likelihood phylogram of the VP35 gene amino acid sequences from filoviruses and related mammalian genomic elements. Numbers on branches represent support values (approximate likelihood ratio tests). Parentheses contain GenBank Accession numbers or museum voucher numbers. Red filled branches indicate placental mammal sequences, blue filled branches indicate marsupial mammal sequences and black filled lines indicate viral sequences. Shaded boxes show the two major clades.

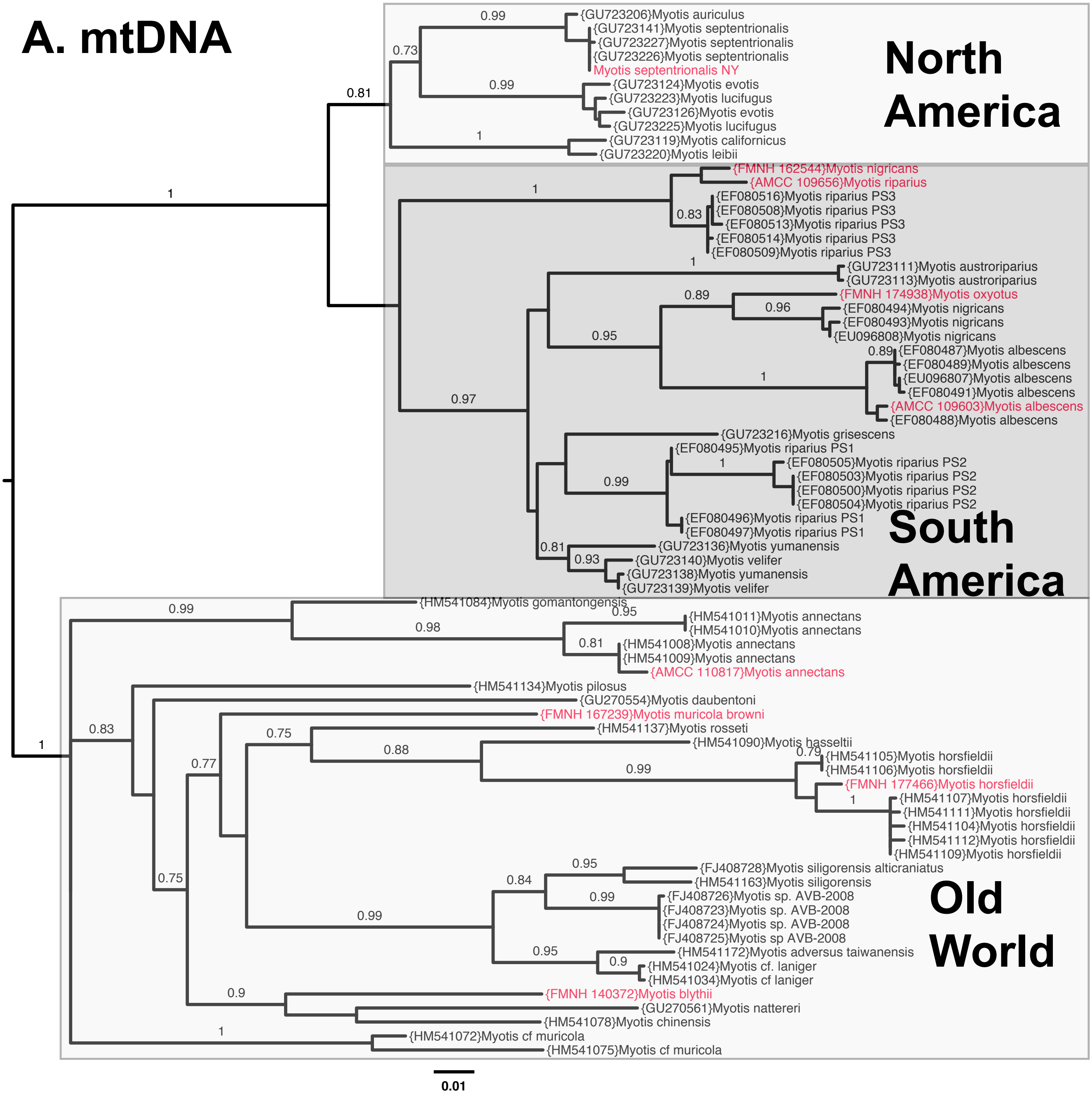
A. mtDNA
North America
South America
Old World
{GU723206}Myotis auriculus
{GU723141}Myotis septentrionalis
{GU723227}Myotis septentrionalis
{GU723226}Myotis septentrionalis
Myotis septentrionalis NY
{GU723124}Myotis evotis
{GU723223}Myotis lucifugus
{GU723126}Myotis evotis
{GU723225}Myotis lucifugus
{GU723119}Myotis californicus
{GU723220}Myotis leibii
{FMNH 162544}Myotis nigricans
{AMCC 109656}Myotis riparius
{EF080516}Myotis riparius PS3
{EF080508}Myotis riparius PS3
{EF080513}Myotis riparius PS3
{EF080514}Myotis riparius PS3
{EF080509}Myotis riparius PS3
{GU723111}Myotis austroriparius
{GU723113}Myotis austroriparius
{FMNH 174938}Myotis oxyotus
{EF080494}Myotis nigricans
{EF080493}Myotis nigricans
{EU096808}Myotis nigricans
{EF080487}Myotis albescens
{EF080489}Myotis albescens
{EU096807}Myotis albescens
{EF080491}Myotis albescens
{AMCC 109603}Myotis albescens
{EF080488}Myotis albescens
{GU723216}Myotis grisescens
{EF080495}Myotis riparius PS1
{EF080505}Myotis riparius PS2
{EF080503}Myotis riparius PS2
{EF080500}Myotis riparius PS2
{EF080504}Myotis riparius PS2
{EF080496}Myotis riparius PS1
{EF080497}Myotis riparius PS1
{GU723136}Myotis yumanensis
{GU723140}Myotis velifer
{GU723138}Myotis yumanensis
{GU723139}Myotis velifer
{HM541084}Myotis gomantongensis
{HM541011}Myotis annectans
{HM541010}Myotis annectans
{HM541008}Myotis annectans
{HM541009}Myotis annectans
{AMCC 110817}Myotis annectans
{HM541134}Myotis pilosus
{GU270554}Myotis daubentoni
{FMNH 167239}Myotis muricola browni
{HM541137}Myotis rosseti
{HM541090}Myotis hasseltii
{HM541105}Myotis horsfieldii
{HM541106}Myotis horsfieldii
{FMNH 177466}Myotis horsfieldii
{HM541107}Myotis horsfieldii
{HM541111}Myotis horsfieldii
{HM541104}Myotis horsfieldii
{HM541112}Myotis horsfieldii
{HM541109}Myotis horsfieldii
{FJ408728}Myotis siligorensis alticraniatus
{HM541163}Myotis siligorensis
{FJ408726}Myotis sp. AVB-2008
{FJ408723}Myotis sp. AVB-2008
{FJ408724}Myotis sp. AVB-2008
{FJ408725}Myotis sp AVB-2008
{HM541172}Myotis adversus taiwanensis
{HM541024}Myotis cf. laniger
{HM541034}Myotis cf laniger
{FMNH 140372}Myotis blythii
{GU270561}Myotis nattereri
{HM541078}Myotis chinensis
{HM541072}Myotis cf muricola
{HM541075}Myotis cf muricola
0.01

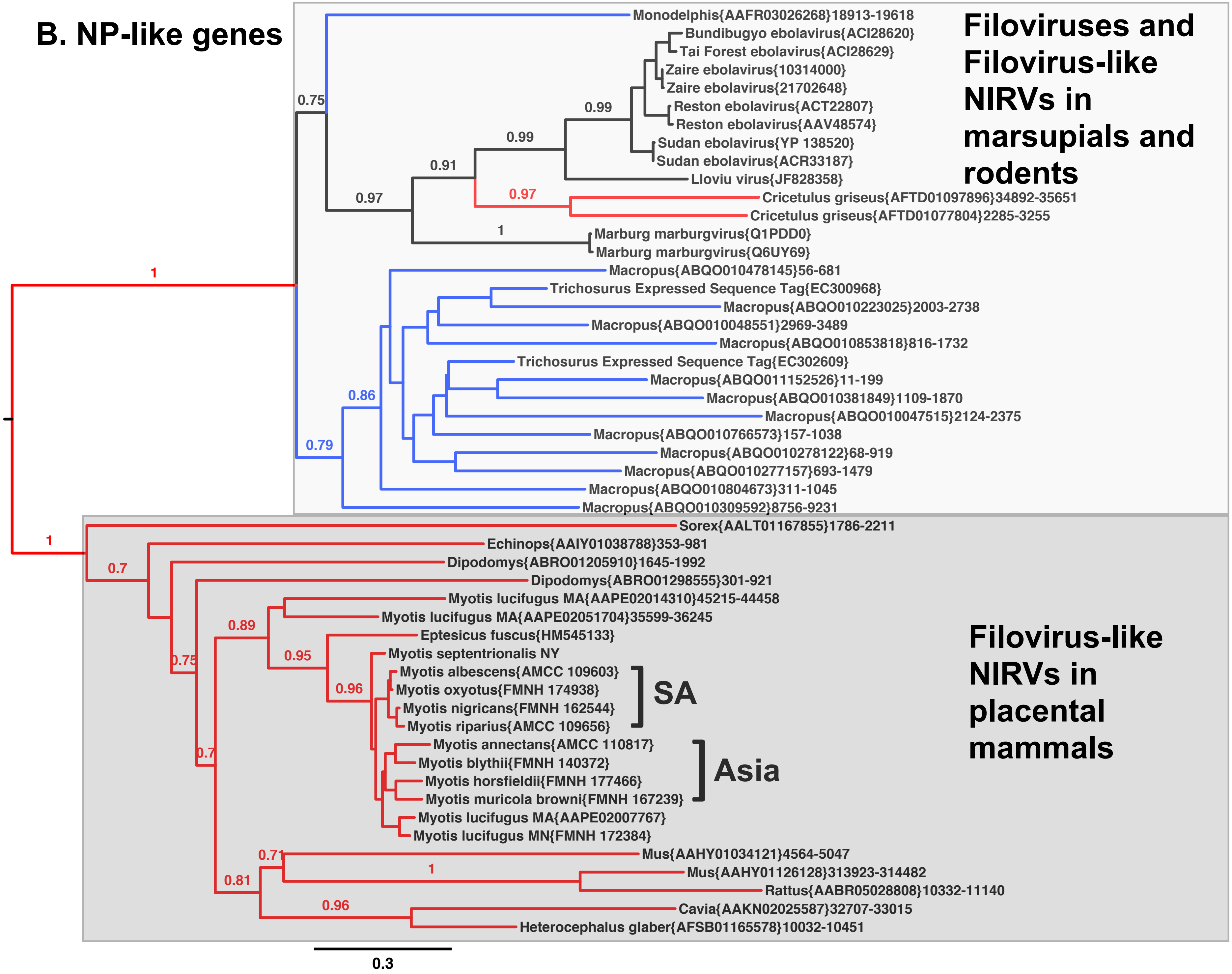
B. NP-like genes
Filoviruses and Filovirus-like NIRVs in marsupials and rodents
Monodelphis{AAFR03026268}18913-19618
Bundibugyo ebolavirus{ACI28620}
Tai Forest ebolavirus{ACI28629}
Zaire ebolavirus{10314000}
Zaire ebolavirus{21702648}
Reston ebolavirus{ACT22807}
Reston ebolavirus{AAV48574}
Sudan ebolavirus{YP 138520}
Sudan ebolavirus{ACR33187}
Lloviu virus{JF828358}
Cricetulus griseus{AFTD01097896}34892-35651
Cricetulus griseus{AFTD01077804}2285-3255
Marburg marburgvirus{Q1PDD0}
Marburg marburgvirus{Q6UY69}
Macropus{ABQO010478145}56-681
Trichosurus Expressed Sequence Tag{EC300968}
Macropus{ABQO010223025}2003-2738
Macropus{ABQO010048551}2969-3489
Macropus{ABQO010853818}816-1732
Trichosurus Expressed Sequence Tag{EC302609}
Macropus{ABQO011152526}11-199
Macropus{ABQO010381849}1109-1870
Macropus{ABQO010047515}2124-2375
Macropus{ABQO010766573}157-1038
Macropus{ABQO010278122}68-919
Macropus{ABQO010277157}693-1479
Macropus{ABQO010804673}311-1045
Macropus{ABQO010309592}8756-9231
Filovirus-like NIRVs in placental mammals
Sorex{AALT01167855}1786-2211
Echinops{AAIY01038788}353-981
Dipodomys{ABRO01205910}1645-1992
Dipodomys{ABRO01298555}301-921
Myotis lucifugus MA{AAPE02014310}45215-44458
Myotis lucifugus MA{AAPE02051704}35599-36245
Eptesicus fuscus{HM545133}
Myotis septentrionalis NY
Myotis albescens{AMCC 109603}
Myotis oxyotus{FMNH 174938}
Myotis nigricans{FMNH 162544}
Myotis riparius{AMCC 109656}
SA
Myotis annectans{AMCC 110817}
Myotis blythii{FMNH 140372}
Myotis horsfieldii{FMNH 177466}
Myotis muricola browni{FMNH 167239}
Asia
Myotis lucifugus MA{AAPE02007767}
Myotis lucifugus MN{FMNH 172384}
Mus{AAHY01034121}4564-5047
Mus{AAHY01126128}313923-314482
Rattus{AABR05028808}10332-11140
Cavia{AAKN02025587}32707-33015
Heterocephalus glaber{AFSB01165578}10032-10451
0.75
0.99
0.99
0.91
0.97
0.97
1
1
0.86
0.79
1
0.7
0.75
0.89
0.95
0.96
0.7
0.71
1
0.81
0.96
0.3

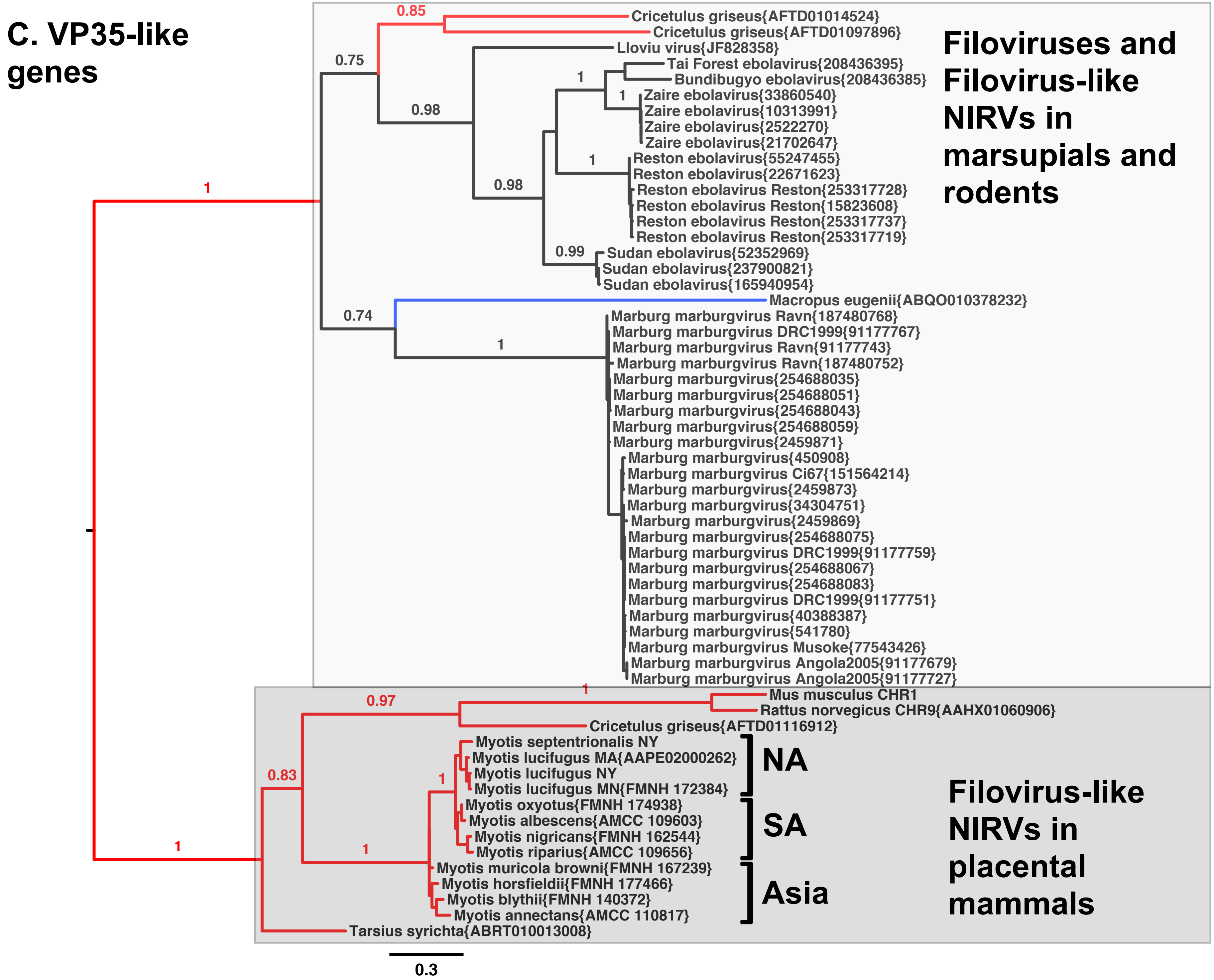
C. VP35-like genes
Filoviruses and Filovirus-like NIRVs in marsupials and rodents
Filovirus-like NIRVs in placental mammals
Cricetulus griseus{AFTD01014524}
Cricetulus griseus{AFTD01097896}
Lloviu virus{JF828358}
Tai Forest ebolavirus{208436395}
Bundibugyo ebolavirus{208436385}
Zaire ebolavirus{33860540}
Zaire ebolavirus{10313991}
Zaire ebolavirus{2522270}
Zaire ebolavirus{21702647}
Reston ebolavirus{55247455}
Reston ebolavirus{22671623}
Reston ebolavirus Reston{253317728}
Reston ebolavirus Reston{15823608}
Reston ebolavirus Reston{253317737}
Reston ebolavirus Reston{253317719}
Sudan ebolavirus{52352969}
Sudan ebolavirus{237900821}
Sudan ebolavirus{165940954}
Macropus eugenii{ABQO010378232}
Marburg marburgvirus Ravn{187480768}
Marburg marburgvirus DRC1999{91177767}
Marburg marburgvirus Ravn{91177743}
Marburg marburgvirus Ravn{187480752}
Marburg marburgvirus{254688035}
Marburg marburgvirus{254688051}
Marburg marburgvirus{254688043}
Marburg marburgvirus{254688059}
Marburg marburgvirus{2459871}
Marburg marburgvirus{450908}
Marburg marburgvirus Ci67{151564214}
Marburg marburgvirus{2459873}
Marburg marburgvirus{34304751}
Marburg marburgvirus{2459869}
Marburg marburgvirus{254688075}
Marburg marburgvirus DRC1999{91177759}
Marburg marburgvirus{254688067}
Marburg marburgvirus{254688083}
Marburg marburgvirus DRC1999{91177751}
Marburg marburgvirus{40388387}
Marburg marburgvirus{541780}
Marburg marburgvirus Musoke{77543426}
Marburg marburgvirus Angola2005{91177679}
Marburg marburgvirus Angola2005{91177727}
Mus musculus CHR1
Rattus norvegicus CHR9{AAHX01060906}
Cricetulus griseus{AFTD01116912}
Myotis septentrionalis NY
Myotis lucifugus MA{AAPE02000262}
Myotis lucifugus NY
Myotis lucifugus MN{FMNH 172384}
Myotis oxyotus{FMNH 174938}
Myotis albescens{AMCC 109603}
Myotis nigricans{FMNH 162544}
Myotis riparius{AMCC 109656}
Myotis muricola browni{FMNH 167239}
Myotis horsfieldii{FMNH 177466}
Myotis blythii{FMNH 140372}
Myotis annectans{AMCC 110817}
Tarsius syrichta{ABRT010013008}
NA
SA
Asia
0.85
0.75
0.98
1
1
1
0.98
0.99
0.74
1
1
0.97
1
0.83
1
1
1
0.3
