# Supplementary material for: Evolutionary maintenance of filovirus-like genes in bat genomes
Source: BMC Evol Biol. 2011 Nov 17;11:336. doi: 10.1186/1471-2148-11-336 (PMC3229293; doi:10.1186/1471-2148-11-336)
Supplement: Additional file 4 — Figure S3. Alignment of filovirus VP35-like nucleotide sequences isolated from bat genomes showing open reading frames. A graphical alignment of the VP35-like region in Myotis with open reading frames followed by a FASTA formatted alignment. Note that the two large indels fail to disrupt the open reading frames. [file 1471-2148-11-336-S4.PDF]

Fig. S3. Alignment of filovirus VP35-like nucleotide sequences isolated from bat genomes. IUPAC ambiguity codes are used for presumed heterozygous sites. A formatted alignment is followed by a FASTA format alignment. The VP35-like region is an open reading frame shown by colored codons. Note that the two indels fail to disrupt the open reading frame.

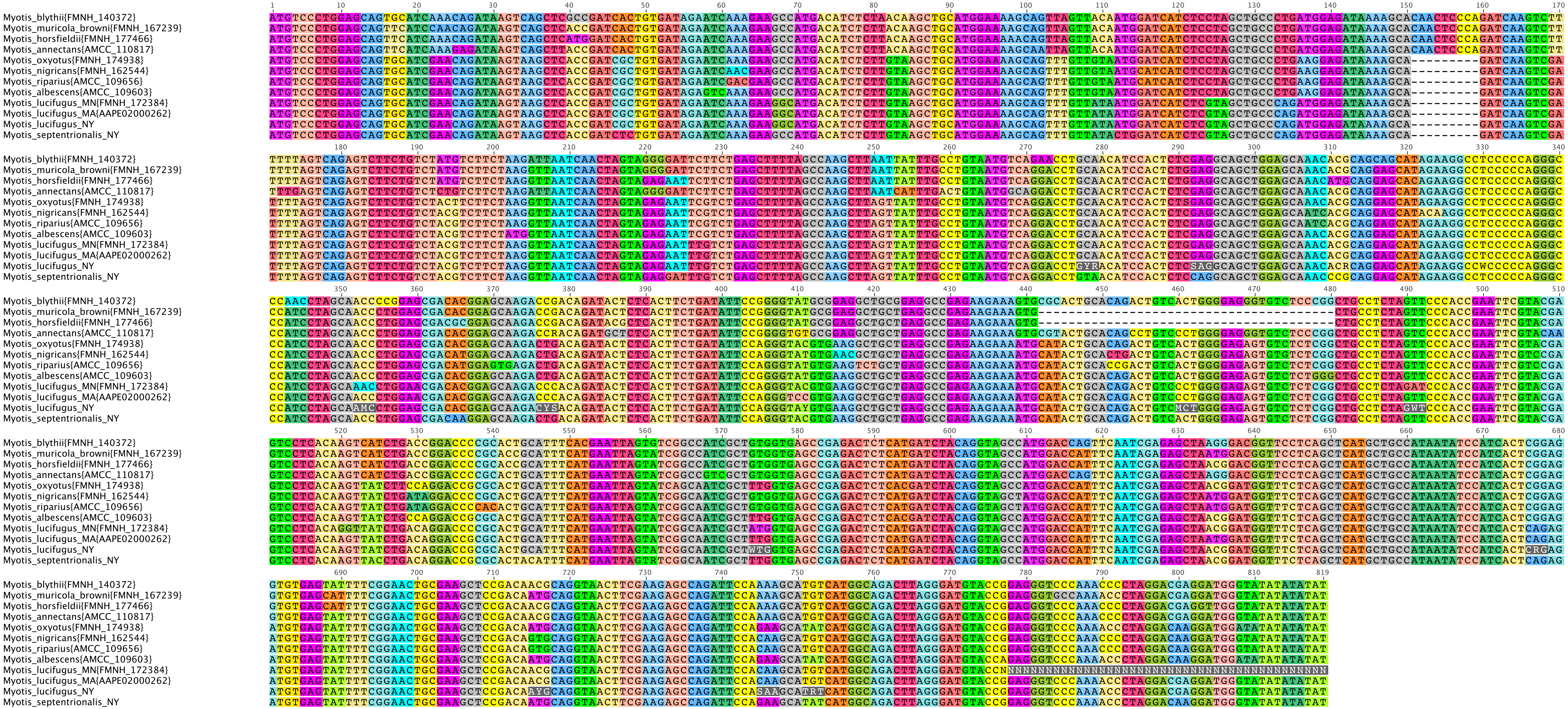
Myotis_blythii{FMNH_140372}
Myotis_muricola_browni{FMNH_167239}
Myotis_horsfieldii{FMNH_177466}
Myotis_annectans{AMCC_110817}
Myotis_oxyotus{FMNH_174938}
Myotis_nigricans{FMNH_162544}
Myotis_riparius{AMCC_109656}
Myotis_albescens{AMCC_109603}
Myotis_lucifugus_MN{FMNH_172384}
Myotis_lucifugus_MA{AAPE02000262}
Myotis_lucifugus_NY
Myotis_septentrionalis_NY

>Myotis_blythii{FMNH_140372}

ATGTCCCTGGAGCAGTGCATCAAACAGATAAGTCAGCTCGCCGATCACTGTGATAGAATCAA
AGAAGCCATGACATCTCT

AACAAGCTGCATGGAAAAGCAGTTAGTTACAATGGATCATCTCCTAGCTGCCCTGATGGAGAT
AAAAGCACAACTCCCAG

ATCAAGTCTTTTTTAGTCAGAGTCTTCTGTCTATGTCTTCTAAGATTAATCAACTAGTAGGGGA
TTCTTCTGAGCTTTTA

GCCAAGCTTAATTATTTGCCTGTAATGTCAGAACCTGCAACATCCACTCTCGAGGCAGCTGGA
GCAAACACGCAGCAGCA

TAGAAGGCCTCCCCCAGGGCCCAACCTAGCAACCCCGGAGCGACACGGAGCAAGACCGACAG
ATACTCTCACTTCTGATA

TTCCGGGGTATGCGGAGGCTGCGGAGGCCGAGAAGAAAGTGCGCACTGCACAGACTGTCACT
GGGGAGGGTGTCTCCCGG

CTGCCTCTAGTTCCCACCGAATTCGTACGAGTCCTCACAAGTCATCTGACCGGACCCCGCACT
GCATTTCACGAATTAGT

GTCGGCCATCGCTGTGGTGAGCCGAGACTCTCATGATCTACAGGTAGCCATGGACCAGTTCAA
TCGAGAGCTAAGGGACG

GTTCCTCAGCTCATGCTGCCATAATATCCATCACTCGGAGGTGTGAGTATTTTCGGAACTGCG
AAGCTCCGACAACGCAG

GTAACTTCGAAGAGCCAGATTCCAAAAGCATGTCATGGCAGACTTAGGGATGTACCGGAGGG
TCCCAAACCCCTAGGACG

AGGATGGGTATATATATAT

>Myotis_muricola_browni{FMNH_167239}

ATGTCCCTGGAGCAGTTCATCCAACAGATAAGTCAGCTCACCGATCACTGTGATAGAATCAAA
GAAGCCATGACATCTCT

TACAAGCTGCATGGAAAAGCAGTTAGTTACAATGGATCATCTCCTCGCTGCCCTGATGGAGAT
AAAAGCACAACTCCCAG

ATCAAGTCTTTTTTAGTCAGAGTCTTCTGTCTATGTCTTCTAAGGTTAATCAACTAGTAGGGGA
TTCTTCTGAGCTTTTA

GCCAAGCTTAATTATTTGCCTGTAATGTCAGGACCTGCAACATCCACTCTGGAGGCAGCTGGA
GCAAACACGCAGGAGCA

TAGAAGGCCTCCCCCAGGGCCCATCCTAGCAACCCTGGAGCGACACGGAGCAAGACCGACAG
ATACTCTCACTTCTGATA

TTCCGGGGTATGCGGAGGCTGCTGAGGCCGAGAAGAAAGTG--------------------------------------

CTGCCTCTAGTTCCCACCGAATTCGTACGAGTCCTCACAAGTCATCTGACCGGACCCCGCACC
GCATTTCATGAATTAGT

ATCGGCCATCGCTGTGGTGAGCCGAGACTCTCATGATCTACAGGTAGCCATGGACCATTTCAA
TAGAGAGCTAATGGACG

GTTCCTCAGCTCATGCTGCCATAATATCCATCACTCGGAGGTGTGAGCATTTTCGGAACTGCG
AAGCTCCGACAATGCAG

GTAACTTCGAAGAGCCAGATTCCAAAAGCATGTCATGGCAGACTTAGGGATGTACCGGAGGG
TGCCAAAACCCTAGGACG

AGGATGGGTATATATATAT

>Myotis_horsfieldii{FMNH_177466}

ATGTCCCTGGAGCAGTTCATCAAACAGATAAGTCAGCTCATGGATCACTGTGATAGAATCAAA
GAAGCCATGACATCTCT

TACAAGCTGCATGGAAAAACAGTTAGTTACAATGGATCATCTCCTAGCTGCCCTGATGGAGAT
AAAAGCACAACTCCCAG

ATCAAGTCTTTTTAGTCAGAGTCTTCTGTCTATGTCTTCTAAGGTTAATCAACTAGTAGAGAA
TTCTTCTGAGCTTTTA

GCCAAGCTTAATTATTTGCCTGTAATGTCAGGACCTGCAACATCCACTCTGGAGGCAGCTGGA
GCAAACATGCAGGAGCA

CAGAAGGCCTCCCCCAGGGCCCATCCTAGCAACCCTGGAGCGACGCGGAGCAAGACCGACAG
ATACGCTCACTTCTGATA

TTCCGGGGTATGCGGAGGCTGCTGAGGCCGAGAAGAAAGTG--------------------------------------

CTGCCTCTAGTTCCCACCGAATTCGTACGAGTCCTCACAAGTCATCTGACCGGACCCCGCACC
GCATTTCATGAATTAGT

ATCGGCCATCGCTGTGGTGAGCCGAGACTCTCATGATCTACAGGTAGCCATGGACCATTTCAA
TCGAGAGCTAACGGACG

GTTCCTCAGCTCATGCTGCCATAATATCCATCACTCGGAGGTGTGAGCATTTTCGGAACTGCG
AAGCTCCGACAACGCAG

GTAACTTCGAAGAGCCAGATTCCAAAAGCATGTCATGGCAGACTTAGGGATGTACCGGAGGG
TCCCAAACCCCTAGGACG

AGGTTGGGTATATATATAT

>Myotis_annectans{AMCC_110817}

ATGTCCCTGGAGCAGTTCATCAAAGAGATAAGTCAGCTTACCGATCACTGTGATAGAATCAAA
GAAGCCATGACATCTCT

TACAAGCTGCATGGAAAAGCAATTAGTTACAATGGATCATCTCCTAGCTGCCCTGATGGAGAT
AAAAGCACAACTCCCAG

ATCAAGTCTTTTTGAGTCAGAGTCTTCTGTCTCTGTCTTCTAAGATTAATCAACTAGTAGGGGA
TTCTTCTGAGCTTTTA

GCCAAGCTTAATCATTTGACTGTAATGGCAGGACCTGCAACRTCCACTCTCGAGGCAGCTGGA
GCAAACACGCAGGAGCA

TAGAAGGCCTCCCCCAGGGCCCATCCTAGCAACCCTGGAGCGACACGGAGCAAGACCRACAG
ATGCTCTCACTTCTGATA

TTCCGGGGTGTGCGGAGGCTGCTGAGGCCGAGAAGAAAGTGCGTACTGCACAGCCTGTCCCT
GGGGAGGGTGTCTCCCGG

CTGCCTCTAGTCCCCACCGAATTCGTACAAGTCCTCACAAGTCATCTGACCGGACCCCGCACT
GCATTTCATGAATTAGT

ATCGGCCGTCGCTGTGGTGAGCCGAGACTCTCATGATCTACAGGTAGCCATGGACCAGTTCAA
TCGAGAGCTAAGGGACG

GTTCCTCAGCTCATGCTGCCATAATATCCATCACTCGGAGGTGTGAGTATTTTCGGAACTGCG
AAGCTCCGACAACGCAG

GTAACTTCGAAGAGCCAGATTCCAAAAGCATGTCATGGCAGACTTAGGGATGTACCGGAGGG
TCCCAAACCCCTAGGACG

AGGATGGGTATATATATAT

>Myotis_oxyotus{FMNH_174938}

ATGTCCCTGGAGCAGTGCATCGAACAGATAAGTAAGCTCACCGATCGCTGTGATAGAATCAA
AGAAGCCATGACATCTCT

TGTAAGCTGCATGGAAAAGCAGTTTGTTGTAATGGATCATCTCCTAGCTGCCCTGAAGGAGAT
AAAAGCA---------G

ATCAAGTCGATTTTAGTCAGAGTCTTCTGTCTACTTCTTCTAAGGTTAATCAACTAGTAGAGAA
TTCGTCTGAGCTTTTA

GCCAAGCTTAGTTATTTGCCTGTAATGTCAGGACCTGCAACATCCACTCTSGAGGCAGCTGGA
GCAAACACGCAGGAGCA

TAGAAGGCCTCCCCCAGGGCCCATCCTAGCAACCCTGGAGCGACACGGAGCAAGACTGACAG
ATACTCTCACTTCTGATA

TTCCAGGGTACGTGAAGGCTGCTGAGGCCGAGAAGAAAATGCATACTGCACAGACTGTCACT
GGGGAGAGTGTCTCTCGG

CTGCCTCTAGTTCCCACCGAATTCGTACGAGTCCTCACAAGTTATCTTCCAGGACCGCGCACTG
CATTTCATGAATTAGT

ATCAGCAATCGCTTTGGTGAGCCGAGACTCTCACGATCTACAGGTAGCCATGGACCATTTCAA
TCGAGAGCTAACGGATG

GTTTCTCAGCTCATGCTGCCATAATATCCATCACTCGGAGATGTGAGTATTTTCGGAACTGCGA
AGCTCCGACAATGCAG

GTAACTTCGAAGAGCCAGATTCCAGAAGCATATCATGGCAGACTTAGGGATGTACCGGAGGG
TCCCAAAACCCTAGGACA

AGGATGGATATATATATAT

>Myotis_nigricans{FMNH_162544}

ATGTCCCTGGAGCAGTGCATCGAACAGATAAGTAAGCTCACCGATCGCTGTGATAGAATCAA
CGAAGCCATGACATCTCT

TGTAAGCTGCATGGAAAAGCAGTTTGTTGTAATGCATCATCTCCTAGCTGCCCTGAAGGAGAT
AAAAGCA---------G

ATCAAGTCGATTTTAGTCAGAGTCTTCTGTCTACGTCTTCTAAGGTTAATCAACTAGTAGAGAA
TTCGTCTGAGCTTTTA

GCCAAGCTTAGTTATTTGCCTGTAATGTCAGGACCTGCAACATCCACTCTCGAGGCAGCTGGA
GCAATCACGCAGGAGCA

TACAAGGCCTCCCCCAGGGCCCATCCTAGCAACCCTGGAGCGACATGGAGCAAGACTGACAG
ATACTCTCACTTCTGATA

TTCCAGGGTATGTGAACGCTGCTGAGGCCGAGAAGAAAATGCATACTGCACTGACTGTCACTG
GGGAGAGTGTGTCTCGG

CTGCCTCTAGTTCCCACCGAATTCGTCCGAGTCCTCACAAGTTATCTGATAGGACCCCGCACTG
CATTTCATGAATTAGT

ATCGGCAATCGCTGTGGTGAGCCGAGACTCTCATGATCTACAGGTAGCTATGGACCATTTCAA
TCGAGAGCTAATGGATG

GTTTCTCAGCTCATGCTGCCATAATATCCATCACTCGGAGATGTGAGTATTTTCGGAACTGCGA
AGCTCCGACAGTGCAG

GTAACTTCGAAGAGCCAGATTCCACAAGCATGTCATGGCAGACTTAGGGATGTACCGGAGGG
TCCCAAACCCCTAGGACA

AGGATGGGTATATATATAT

>Myotis_riparius{AMCC_109656}

ATGTCCCTGGAGCAGTGCATCGAACAGATAAGTAAGCTCACCGATCGCTGTGATAGAATCGA
CGAAGCCATGACATCTCT

TGTAAGCTGCATGGAAAAGCAGTTTGTTGTAATGCATCATCTCCTAGCTGCCCTGAAGGAGAT
AAAAGCA---------G

ATCAAGTCGATTTTAGTCAGAGTCTTCTGTCTACGTCTTCTAAGGTTAATCAACTAGTAGAGAA
TTCGTCTGAGCTTTTA

GCCAAGCTTAGTTATTTGCCTGTAATGTCAGGACCTGCAACATCCACTCTCGAGGCAGCTGGA
GCAATCACGCAGGAGCA

TAGAAGGCCTCCCCCAGGGCCCATCCTAGCAACCCTGGAGCGACATGGAGTGAGACTGACAG
ATACTCTCACTTCTGATA

TTCCAGGGTATGTGAAGTCTGCTGAGGCCGAGAAGAAAATGCATACTGCACCGACTGTCACTG
GGGAGAGTGTCTCTCGG

CTGCCTCTAGTTCCCACCGAATTCGTCCGAGTCCTCACAAGTTATCTGATAGGACCCCACACTG
CATTTCATGAATTAGT

ATCGGCAATCGCTGTGGTGAGCCGAGACTCTCATGATCTACAGGTAGCTATGGACCATTTCAA
TCGAGAGCTAATGGATG

GTTTCTCAGCTCATGCTGCCATAATATCCATCACTCGGAGATGTGAGTATTTTCGGAACTGCGA
AGCTCCGACAGTGCAG

GTAACTTCGAAGAGCCAGATTCCACAAGCATGTCATGGCAGACTTAGGGATGTACCGGAGGG
TCCCAAACCCCTAGGACA

AGGATGGGTATATATATAT

>Myotis_albescens{AMCC_109603}

ATGTCCCTGGAGCAGTGCATCGAACAGATAAGTAAGCTCACCGATCGCTGTGATAGAGTCAA
AGAAGCCATGACATCTCT

TGTAAGCTGCATGGAAAAGCAGTTTGTTGTAATGGATCATCTCCTAGCTGCCCTGAAGGAGAT
AAAAGCA---------G

ATCAAGTCGATTTTAGTCAGAGTCTTCTGTCTACGTCTTCTATGGTTAATCAACTAGTAGAGAA
TTCGTCTGAGCTTTTA

GCCAAGCTTAGTTATTTGCCTGTAATGTCAGGACCTGCAACATCCACTCTCGAGGCAGCTGGA
GCAAACACGCAGGAGCA

TAGAAGGCCTCCCCCAGGGCCCATCCTAGCAACCCTGGAGCGACACGGAGCAAGACTGACAG
ATACTCTCACTTCTGATA

TTCCGGGGTATGTGAAGGCTGCTGAGGCCGAGAAGAAAATGCATACTGCACAGACTGTCACT
GGGGAGAGTGTCTCTGGG

CTGCCTCTAGTTCCCACCGAATTCGTACGAGTCCTCACAAGTTATCTGCCAGGACCGCGCACT
GCATTTCATGAATTAGT

ATCGGCAATCGCTTTGGTGAGCCGAGACTCTCACGATCTACAGGTAGCCATGGACCATTTCAA
TCGAGAGCTAACGGATG

GTTTCTCAGCTCATGCTGCCATAATATCCATCACTCGGAGATGTGAGTATTTTCGGAACTGCGA
AGCTCCGACAATGCAG

GTAACTTCGAAGAGCCAGATTCCAGAAGCATATCATGGCAGACTTAGGGATGTACCAGAGGG
TCCCAAAACCCTAGGACA

AGGATGGATATATATATAT

>Myotis_lucifugus_MN{FMNH_172384}

ATGTCCCTGGAGCAGTGCATCGAACAGATAAGTAAGCTCACCGATCGCTGTGATAGAATCAA
AGAAGGCATGACATCTCT

TGTAAGCTGCATGGAAAAGCAGTTTGTTATAATGGATCATCTCGTAGCTGCCCAGATGGAGAT
AAAAGCA---------G

ATCAAGTCGATTTTAGTCAGAGTCTTCTGTCTACGTCTTCTAAGGTTAATCAACTAGTAGAGAA
TTTGTCTGAGCTTTTA

GCCAAGCTTAGTTATTTGCCTGTAATGTCAGGACCTGCAACATCCACTCTGGAGGCAGCTGGA
GCAAACACGCAGGAGCA

TAGAAGGCCTCCCCCAGGGCCCATCCTAGCAAACCTGGAACGACACGGAGCAAGACCCACAG
ATACTCTCACTTCTGATA

TTCCAGGGTACGTGAAGGCTGCTGAGGCCGAGAAGAAAATGCATACTGCACAGACTGTCCCT
GGGGAGAGTGTCTCTCGG

CTGCCTCTAGATCCCACCGAATTCGTACGAGTCCTCACAGGTTATCTGACAGGACCCCGCACT
GCATTTCATGAATTAGT

ATCGGCAATCGCTATGGTGAGCCGAGACTCTCATGATCTACAGGTAGCCATGGACCATTTCAA
TCGAGAGCTAACGGATG

GTTTCTCAGCTCATGCTGCCATAATATCCATCACTCAGAGATGTGAGTATTTTCGGAACTGCGA
AGCTCCGACAACGCAG

GTAACTTCGAAGAGCCAGATTCCACAAGCATGTCATGGCAGACTTAGGGATGTACCNNNNNN
NNNNNNNNNNNNNNNNNN

NNNNNNNNNNNNNNNNNNN

>Myotis_lucifugus_MA{AAPE02000262}

ATGTCCCTGGAGCAGTGCATCGAACAGATAAGTAAGCTCACCGATCGCTGTGATAGAATCAA
AGAAGGCATGACATCTCT

TGTAAGCTGCATGGAAAAGCAGTTTGTTATAATGGATCATCTCGTAGCTGCCCAGATGGAGAT
AAAAGCA---------G

ATCAAGTCGATTTTAGTCAGAGTCTTCTGTCTACGTCTTCTAAGGTTAATCAACTAGTAGAGAA
TTTGTCTGAGCTTTTA

GCCAAGCTTAGTTATTTGCCTGTAATGTCAGGACCTGCAACATCCACTCTCGAGGCAGCTGGA
GCAAACACGCAGGAGCA

TAGAAGGCCTCCCCCAGGGCCCATCCTAGCAACCCTGGAACGACACGGAGCAAGACCCACAG
ATACTCTCACTTCTGATA

TTCCAGGGTCCGTGAAGGCTGCTGAGGCCGAGAAGAAAATGCATACTGCACAGACTGTCCCT
GGGGAGAGTGTCTCTCGG

CTGCCTCTAGTTCCCACCGAATTCGTACGAGTCCTCACAAGTTATCTGACAGGACCGCGCACT
GCATTTCATGAATTAGT

ATCGGCAATCGCTTTGGTGAGCCGAGACTCTCATGATCTACAGGTAGCCATGGACCATTTCAA
TCGAGAGCTAATGGATG

GTTTCTCAGCTCATGCTGCCATAATATCCATCACTCAGAGATGTGAGTATTTTCGGAACTGCGA
AGCTCCGACAACGCAG

GTAACTTCGAAGAGCCAGATTCCACAAGCATGTCATGGCAGACTTAGGGATGTACCGGAGGG
TCCCAAAACCCTAGGACG

AGGATGGGTATATATATAT

>Myotis_lucifugus_NY

ATGTCCCTGGAGCAGTGCATCGAACAGATAAGTAAGCTCACCGATCGCTGTGATAGAATCAA
AGAAGGCATGACATCTCT

TGTAAGCTGCATGGAAAAGCAGTTTGTTATAATGGATCATCTCGTAGCTGCCCAGATGGAGAT
AAAAGCA---------G

ATCAAGTCGATTTTAGTCAGAGTCTTCTGTCTACGTCTTCTAAGGTTAATCAACTAGTAGAGAA
TTTGTCTGAGCTTTTA

GCCAAGCTTAGTTATTTGCCTGTAATGTCAGGACCTGYRACATCCACTCTCSAGGCAGCTGGA
GCAAACACRCAGGAGCA

TAGAAGGCCWCCCCCAGGGCCCATCCTAGCAAMCCTGGAGCGACACGGAGCAAGACYSACA
GATACTCTCACTTCTGATA

TTCCAGGGTAYGTGAAGGCTGCTGAGGCCGAGAAGAAAATGCATACTGCACAGACTGTCMCT
GGGGAGAGTGTCTCTCGG

CTGCCTCTAGWTCCCACCGAATTCGTACGAGTCCTCACAAGTTATCTGACAGGACCGCGCACT
GCATTTCATGAATTAGT

ATCGGCAATCGCTWTGGTGAGCCGAGACTCTCATGATCTACAGGTAGCCATGGACCATTTCAA
TCGAGAGCTAACGGATG

GTTTCTCAGCTCATGCTGCCATAATATCCATCACTCRGAGATGTGAGTATTTTCGGAACTGCGA
AGCTCCGACAAYGCAG

GTAACTTCGAAGAGCCAGATTCCASAAGCATRTCATGGCAGACTTAGGGATGTACCGGAGGGT
CCCAAAACCCTAGGACG

AGGATGGGTATATATATAT

>Myotis_septentrionalis_NY

ATGTCCCTGGAGCAGTGCATCGAACAGATAAGTAAGCTCACCGATCTCTGTGATAGAATCAAA
GAAGCCATGACATCTCT

TGTAAGCTGCATGGAAAAGCAGTTTGTTATACTGGATCATCTCGTAGCTGCCCAGATGGAGAT
AAAAGCA---------G

ATCAAGTCGATTTTAGTCAGAGTCTTCTGTCTACGTCTTCTAAGGTTAATCAACTAGTAGAGGA
TTTGTCTGAGCTTTTA

GCCAAGCTTAGTTATTTGCCTGTAATGTCAGGACCTGTAACATCCACTCTCCAGGCAGCTGGA
GCAAACCCGCAGGAGCA

TAGAAGGCCTCCCCCAGGGCCCATCCTAGCAACCCTGGAGCGACAAGGAGCAAGACTGACAG
ATACTCTCACTTCTGATA

TTCCAGGGTACGTGAAGGCTGCTGAGGCCGAGAAGAAAATGCATACTGCACAGACTGTCACT
GGGGAGAGTGTCTCTCGG

CTGCCTCTAGTTCCCACCGAATTCGTACGAGTCCTCACAAGTTACCTGACAGGACCGCGCACT
ACATTTCATGAATTAGT

RTCGGCAATCGCTTTGGTGAGCCGAGACTCTCATGATCTACAGGTAGCCATGGACCATTTCAA
TCGAGAGCTAACGGATG

GTTTCTCAGCTCATGCTGCCATAATATCCATCACTCAGAGATGTGAGTATTTTCGGAACTGCGA
AGCTCCGACAATGCAG

GTAACTTCGAAGAGCCAGATTCCAGAAGCATATCATGGCAGACTTAGGGATGTACCGGAGGG
TCCCAAAACCCTAGGACA

AGGATGGGTATATATATAT
