# Supplementary material for: Evolutionary maintenance of filovirus-like genes in bat genomes
Source: BMC Evol Biol. 2011 Nov 17;11:336. doi: 10.1186/1471-2148-11-336 (PMC3229293; doi:10.1186/1471-2148-11-336)
Supplement: Additional file 5 — Figure S4. Alignment of filovirus nucleoprotein (NP)-like nucleotide sequences isolated from bat genomes. A graphical alignment of the NP-like region in Myotis followed by a FASTA formatted alignment. [file 1471-2148-11-336-S5.PDF]

Fig. S4. Alignment of filovirus nucleoprotein (NP)-like nucleotide sequences isolated from bat genomes. IUPAC ambiguity codes are used for presumed heterozygous sites. A formatted alignment is followed by a FASTA format alignment.

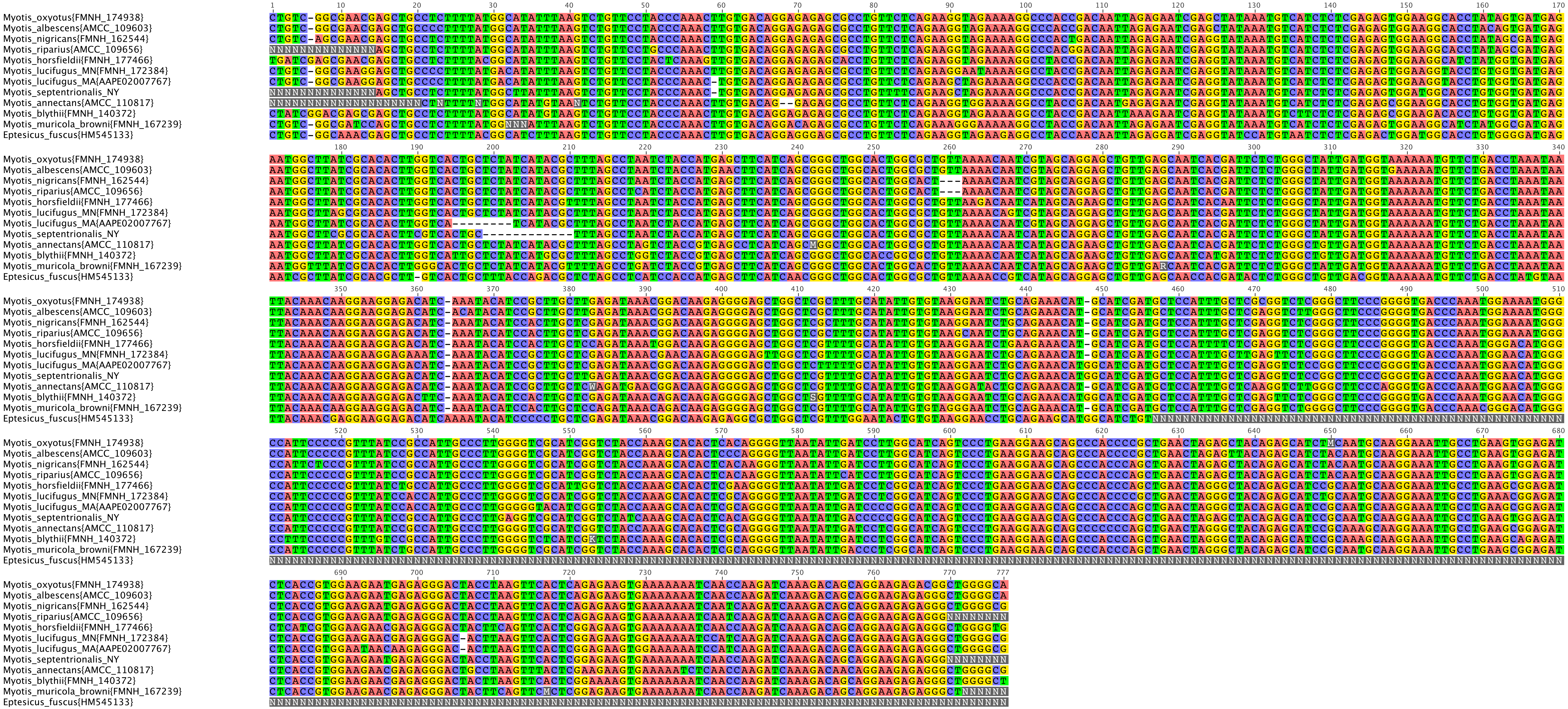
Myotis_oxyotus{FMNH_174938}
Myotis_albescens{AMCC_109603}
Myotis_nigricans{FMNH_162544}
Myotis_riparius{AMCC_109656}
Myotis_horsfieldii{FMNH_177466}
Myotis_lucifugus_MN{FMNH_172384}
Myotis_lucifugus_MA{AAPE02007767}
Myotis_septentrionalis_NY
Myotis_annectans{AMCC_110817}
Myotis_blythii{FMNH_140372}
Myotis_muricola_browni{FMNH_167239}
Eptesicus_fuscus{HM545133}

>Myotis_oxyotus{FMNH_174938}

CTGTC-
GGCGAACGAGCTGCCTCTTTTATGGCATATTTAAGTCTGTTCCTACCCAAACTTGTGA
CAGGAGAGAGCGCCTGTTCTCAGAAGGTAGAAAAGGCCCACCGACAATTAGAGAAT
CGAGCTATAAATGTCATCTCTCGAGAGTGGAAGGCACCTATAGTGATGAGAATGGCT
TATCGCACACTTGGTCACTGCTCTATCATACGCTTTAGCCTAATCTACCATGAGCTTC
ATCAGCGGGCTGGCACTGGCGCTGTTAAAACAATCGTAGCAGGAGCTGTTGAGCAAT
CACGATTCTCTGGGCTATTGATGGTAAAAAATGTTCTGACCTAAATAATTACAAACA
AGGAAGGAGACATC-
AAATACATCCGCTTGCTTGAGATAAACGGACAAGAGGGGAGCTGGCTCGCTTTGCAT
ATTGTGTAAGGAATCTGCAGAAACAT-
GCATCGATGCTCCATTTGCTCGCGGTCTCGGGCTTCCCGGGGTGACCCAAATGGAAA
ATGGGCCATTCCCCCGTTTATCCGCCATTGCCCTTGGGGTCGCATCGGTCTACCAAAG
CACACTCACAGGGGTTAATATTGATCCTTGGCATCAGTCCCTGAAGGAAGCAGCCCA
CCCCGCTGAACTAGAGCTACAGAGCATCTMCAATGCAAGGAAATTGCCTGAAGTGG
AGATCTCACCGTGGAAGAATGAGAGGGACTACCTAAGTTCACTCAGAGAAGTGAAA
AAAATCAACCAAGATCAAAGACAGCAGGAAGAGACGGCTGGGGCA

>Myotis_albescens{AMCC_109603}

CTGTC-
GGCGAACGAGCTGCCCCTTTTATGGCATATTTAAGTCTGTTCCTACCCAAACTTGTGA
CAGGAGAGAGCGCCTGTTCTCAGAAGGTAGAAAAGGCCCACCGACAATTAGAGAAT
CGAGCTATAAATGTCATCTCTCGAGAGTGGAAGGCACCTACAGTGATGAGAATGGCT
TATCGCACACTTGGTCACTGCTCTATCATACGCTTTAGCCTAATCTACCATGAACTTC
ATCAGCGGGCTGGCACTGGCGCTGTTAAAACAATCGTAGCAGGAGCTGTTGAGCAAT
CACGATTCTCTGGGCTATTGATGGTGAAAAATGTTCTGACCTAAATAATTACAAACA
AGGAAGGAGACATC-
ACATACATCCGCTTGCTTGAGATAAACGGACAAGAGGGGAGCTGGCTCGCTTTGCAT
ATTGTGTAAGGAATCTGCAGAAACAT-
GCATCGATGCTCCATTTGCTCGAGGTCTTGGGCTTCCCGGGGTGACCCAAATGGAAA
ATGGGCCATTCCCCCGTTTATCCGCCATTGCCCTTGGGGTCGCATCGGTCTACCAAAG
CACACTCCCAGGGGTTAATATTGATCCTTGGCATCAGTCCCTGAAGGAAGCAGCCCA
CCCCGCTGAACTAGAGTTACAGAGCATCTACAATGCAAGGAAATTGCCTGAAGTGGA
GATCTCACCGTGGAAGAATGAGAGGGACTACCTAAGTTCACTCAGAGAAGTGAAAA
AAATCAACCAAGATCAAAGACAGCAGGAAGAGAGGGCTGGGGCA

>Myotis_nigricans{FMNH_162544}

CTGTC-
AGCGAACGAGCTGCCTCTTTTATGGCATATTTAAGTCTGTTCCTACCCAAACTTGTGA
CAGGAGAGAGCGCCTGTTCTCAGAAGGTAGAAAAGGCCCACTGACAATTAGAGAAT
CGAGGTATAAATGTCATCTCTCGAGAGTGGAAGGCACCTATAGCGATGAGAATGGCT
TATCGCACACTTGGTCACTGCTCTATCATACGCTTTAGCCTAATCTACCATGAGCTTC
ATCAGCGGGCTGGCACTGGCACT---
AAAACAATCGTAGCAGGAGCTGTTGAGCAATCACGATTCTCTGGGCTATTGATGGTA
AAAAATGTTCTGACCTAAATAATTACAAACAAGGAAGGAGACATC-
AAATACATCCACTTGCTCGAGATAAACGGACAAGAGGGGAGCTGGCTCGCTTTGCAT
ATTGTGTAAGGAATCTGCAGAAACAT-
GCATCGATGCTCCATTTGCTCGAGGTCTCGGGCTTCCCGGGGTGACCCAAATGGAAA
ATGGGCCATTCTCCCGTTTATCCGCCATTGCCCTTGGGGTCGCATCGGTCTACCAAAG

CACACTCACAAGGGTTAATATTGATCCTTGGCATCAGTCCCTGAAGGAAGCAGCCCA
CCCAGCTGAACTAGAGCTACAGAGCATCTACAATGCAAGGAAATTGCCTGAAGTGG
AGATCTCACCGTGGAAGAATGAGAGGGACTACCTAAGTTCACTCAGAGAAGTGAAA
AAAATCAATCAAGATCAAAGACAGCAGGAAGAGAGGGCTGGGGCG

>Myotis_riparius{AMCC_109656}

NNNNNNNNNNNNNNAGCTGCCTCTTTTATGGCATATTTAAGTCTGTTCCTGCCCAAA
CTTGTGACAGGAGAGAGCGCCTGTTCTCAGAAGGTAGAAAAGGCCCACGGACAATT
AGAGAATCGAGGTATAAATGTCATCTCTCGAGAGTGGAAGGCACCTATAGCGATGA
GAATGGCTTATCGCACACTTGGTCACTGCTCTATCATACGCTTTAGCCTCATCTACCA
TGAGCTTCATCAGCGGGCTGGCACTGGCACT---
AAAACAATCGTAGCAGGAGCTGTTGAGCAATCACGATTCTCTGGGCTATTGATGGTA
AAAAATGTTCTGACCTAAATAATTACAAACAAGGAAGGAGACATC-
AAATACATCCACTTGCTCGAGATAAACGGACAAGAGGGGAGCTGGCTCGCTTTGCAT
ATTGTGTAAGCAATCTGCAGAAACAT-
GCATCGATGCTCCATTTGCTCGAGGTCTCGGGCTTCCCGGGGTGACCCAAATGGAAA
ATGGGCCATTCCCCCGTTTATCCGCCATTGCCCTTGGGGTCGCATCGGTCTACCAAAG
CACACTCACAAGGGTTAATATTCATCCTTGGCATCAGTCCCTGAAGGAAGCAGCCCA
CCCAGCTGAACTAGAGCTACAGAGCATCTACAATGCAAGGAAATTGCCTGAAGTGG
AGATCTCACCGTGGAAGAATGAGAGGGACTACCTAAGTTCACTCAGAGAAGTGAAA
AAAATCAATCAAGATCAAAGACAGCAGGAAGAGAGGGNNNNNNNN

>Myotis_horsfieldii{FMNH_177466}

TGATCGAGCGAACGAGCTGCCTCTTTTACGGCATATTTAAGTCTGTTCCTACTCAAAG
TTGTGACAGGAGAGAGCACCTGTTCTCAGAAGGTAGAAAAGGCCTACCGACAATTA
GAGAATCGAGGTATAAATGTCATCTCTCGAGAGTGGAAGGCATCTATGGTGATGAGA
ATGGCTTATCGCACACTTGGTCACTGCTCTATCATACGTTTTAGCCTAATCTACCATG
AGCTTCATCAGCGGGCTGGCACTGGCGCTGTTAAGACAATCATAGCAGAAGCTGTTG
AGCAATCACAATTCTCTGGGCTATTGATGGTAAAAAATGTTCTGACCTAAATAATTA
CAAACAAGGAAGGAGACATC-
AAATACATCCACTTGCTCCAGATAAATGGACAAGAGGGGAGCTGGCTCGTTTTGCAT
ATTGTGTAAGGAATCTGAAGAAACAT-
GCATCGATGCTCCATTTTCTCGAGGTCTCGGGCTTCCCGGGGTGACCCAAATGGGAC
ATGGGCCATTCCCCCGTTTATCTGCCATTGCCCTTGGGGTCGCATTGGTCTACCAAAG
CACACTCGAAGGGGTTAATATTGATCCTCGGCATCAGTCCCTGAAGGAAGCAGCCCA
CCCAGCTGAACTAGGGCTACAGAGCATCCGCAATGCAAGGAAATTGCCTGAAGCGG
AGATCTCATCGTGGAAGAACGAGAGGGACTACTTCAGTTCACTCGGAGAAGTGAAA
AAAATCAACCAAGATCAAAGACAGCAGGAAGAGAGGGCTGGGGTG

>Myotis_lucifugus_MN{FMNH_172384}

CTGTC-
GGCGAAGGAGCTGCCCCTTTTATGACATATTTAAGTCTGTTCCTACCCAAACTTGTGA
CAGGAGAGAGCGCCTGTTCTCAGAAGGAATAAAAGGCCTACCGACAATTAGAGAAT
CGAGGTATAAATGTCATCTCTCGAGAGTGGAAGGTACCTGTGGTGATGAGAATGGCT
TAGCGCACACTTGGTCACTGCTCTATCATACGCTTTAGCCTAATCTACCATGAGCTTC
ATCAGCGGGCTGGCACTGGCGCTGTTAAAACAGTCGTAGCAGGAGCTGTTGAGCAAT
CACGATTCTCTGGGCTATTGATGGTAAAAAATGTTCTGACCTAAATAATTACAAACA
AGGAAGGAGAAATC-
AAATACATCCGCTTGCTCGAGATAAACGAACAAGAGGGGAGTTGGCTCGTTTTGCAT

ATTGTGTAAGGAATCTGCAGAAACAT-
GCATCGATGCTCCATTTGCTTGAGTTCTCGGGCTTCCCGGGGTGACCCAAATGGAAC
ATGGGCCATTCCCCCGTTTATCCACCATTGCCCTTGGGGTCGCATCGGTCTACCAAAG
CACACTCGCAGGGGTTAATATTGATCCTCGGCATCAGTCCCTGAAGGAAGCAGCCCA
CCCCGCTGAACTAGGGCTACAGAGCATCTGCAATGCAAGGAAATTGCCTGAAACGG
AGATCTCACCGTGGAAGAACGAGAGGGAC-
ACTTAAGTTCACTCGGAGAAGTGGAAAAAATCCATCAAGATCAAAGACAGCAGGAA
GAGAGGGCTGGGGCG

>Myotis_lucifugus_MA{AAPE02007767}

CTGTC-GGCGAAGGAGCTGCCCCTTTTATGACATATTTAAGTCTGTTCCTACCCAAAC-
TGTGACAGGAGAGAGCGCCTGTTCTCAGAAGCTAGAAAAGGCCCACCGACAATTAG
AGAATCGAGGTATAAATGTCATCTCTCGAGAGTGGAAGGTACCTGTGGTGATGAGAA
TGGCTTATCGCACACTTGGTCA--------
TCATACGCTTTAGCCTAATCTACCATGAGCTTCATCAGCGGGCTGGCACTGGCGCTGT
TAAAACAATCGTAGCAGGAGCTGTTGAGCAATCACGATTCTCTGGGCTATTGATGGT
AAAAAATGTTCTGACCTAAATAATTACAAACAAGGAAGGAGACATC-
AAATACATCCGCTTGCTCGAGATAAACGGACAAGAGGGGAGCTGGCTCTTTTTGCAT
ATTGTGTAAGGAATCTGCAGAAACATGGCATCGATGCTCCATTTGCTCGAGGTCTCC
GGCTTCCCGGGGTGACCCAAATGGAACATGGGCCATTCCCCCGTTTATCCACCATTG
CCCTTGGGGGTACATCGGTCTACCAAAGCACACTCGCAGGGGTTAATATTGATCCCC
GGCATCAGTCCCTGAAGGAAGCAGCCCACCCAGCTGAACTAGGGCTACAGAGCATC
CGCAATGCAAGGAAATTGCCTGAAGCGGAGATCTCACCGTGGAATAACAAGAGGGA
C-
ACTTAAGTTCACTCGGAGAAGTGGAAAAAATCCATCAAGATCAAAGACAGCAGGAA
GAGAGGGCTGGGGCG

>Myotis_septentrionalis_NY

NNNNNNNNNNNNNNAGCTGCCTCTTTTATGGCTTATTTAAGTCTGTTCCTACCCAAA
C-
TGTGACAGGAGAGAGCGCCTGTTTTCAGAAGCTAGAAAAGGCCCACCGACAATTAG
AGAATCGAGGTATAAATGTCATCTCTCGAGAGTGGATGGCACCTGTGGTGATGAGAA
TGGCTTCGCGCACACTTCGTCACTGC------------
TTTAGCCTAATCTACCATGAGCTTCATCAGCGGGCTGGCACTGGCGCTGTTAAAACA
ATCATAGCAGGAGCTGTTGAGCAATCACGATTCTCTGGGCTATTGATGGTAAAAAAT
GTTCTGACCTAAATAATTACAAACAAGGAAGGAGACATC-
AAATACATCCGCTTGCTTGAGATAAACGGACAAGAGGGGAGCTGGCTCGTTTTGCAT
ATTGTGTAAGGAATCTGCAGAAACATGGCATCGATGCTCCATTTGCTCGAGGTCTCC
GGCTTCCCGGGGTGACCCAAATGGAACATGGGCCATTCCCCCGTTTATCCGCCATTG
CCCTTGAGGTCGCATCGGTCTATCAAAGCACACTCACAGGGGTTAATATTGACCCCC
GGCATCAGTCCCTGAAGGAAGCAGCCCACCCAGCTGAACTAGAGCTACAGAGCATC
CGCAATGCAAGGAAATTGCCTGAAGTGGAGATCTCACCGTGGAAGAATGAGAGGGA
CTACCTAAGTTCACTCGGAGAAGTGAAAAAAATCAACCAAGATCAAAGACAGCAGG
AAGAGAGGGNNNNNNNN

>Myotis_annectans{AMCC_110817}

NNNNNNNNNNNNNNNNNNNCTNTTTTNTGGCATATGTAANTCTGTTCCTACCCAAA
CTTGTGACAG--
GAGAGCGCCTGTTCTCAGAAGGTGGAAAAGGCCTACCGACAATGAGAGAATCGAGG

TATAAATGTCATCTCTCGAGAGCGGAAGGCACCTGTGGTGATGAGAATGGCTTATCG
CACACTTGGTCACTGCTCTATCATACGCTTTAGCCTAGTCTACCGTGAGCCTCATCAG
CMGGCTGGCACTGGCGCTGTTAAAACAATCATAGCAGAAGCTGTTGAGCAATCACG
ATTCTCTGGGCTGTTGATGGTAAAAAATGTTCTGACCTAAATAATTACAAACAAGGA
AGGAGACATC-
AAATACATCCGCTTGCTCWAGATGAACGGACAAGAGGGGAGCTGGCTCGTTTTGCA
TATTGTGTAAGGATACTGCAGAAACAT-
GCATCGATGCTCCATTTGCTCAAGGTCTTGGGCTTCCCAGGGTGACCCAAATGGAAC
ATGGGCCATTCCCCCGTTTATCCGCCATTGCCCTTGGGGTCGCATCGGTCTACCAAAG
CACACTCGCAGGGGTTAATATTGATCCTCGGCATCAGTCCCTGAAGGAAGCAGCCCC
CCCAGCTGAACTAGGGCTACAGAGCATCCGCAAAGCAAGGAAATTGCCTGAAGCGG
AGATCTCACCGTGGAAGAACGAGAGGGACTGCCTAAGTTTACTCGAAGAAGTGAAA
AATCTCAACCAAGATCAAAGACAACAGGAAGAGAGGGCTGGGGCG

>Myotis_blythii{FMNH_140372}

CTATCGGACGAGCGAGCTGCCTCTTTTATGGCATATGTAAGTCTGTTCCTACCCAAAC
TTGTGACAGGAGAGAGCGCCTGTTCTCAGAAGGTAGAAAAGGCCTACCGACAATTA
AAGAATCGAGGTATAAATGTCTTCTCTCGAGAGCGGAAGACACCTGTGGTGATGAGA
ATGGCTTATCGCACACTTGGTCATTGCTCTATCATGCGCTTTAGCCTGGTCTACCGTG
AGCTTCATCAGCGGGCTGGCACCGGCGCTGTTAAAACAATCATAGCAGAAGCTGTTG
AGCAATCATGATTCTCTGGGCTGTTGATGGTAAAAAATGTTCTGACCTAAATAATTA
CAAACAAGGAAGGAGACTTC-
AAATACATCCACTTGCTCGAGATAAACAGACAAGAGGGGAGCTGGCTSGTTTTGCAT
ATTGTGTAAGGAATCTGCAGAAACATGGCATCGATGCTCCATTTGCTCGAGTTCTCG
GGCTTCCCGGGGTGACCCAAATGGAACATGGGCCTTTCCCCCGTTTGTCCGCCATTGC
CCTTGGGGTCTCATCGKTCTACCAAAGCACACTCGCAGGGGTTAATATTGATCCTCG
GCATCAGTCCCTGAAGGAAGCAGCCCACCCAGCTGAACTAGGGCTACAGAGCATCC
GCAAAGCAAGGAAATTGCCTGAAGCAGAGATCTCACCGTGGAAGAACGAGAGGGAC
TACTTAAGTTCACTCGGAAAAGTGAAAAAAATCAACCAAGATCAAAGACAGCAGGA
AGAGAGGGCTGGGGCT

>Myotis_muricola_browni{FMNH_167239}

CTGTC-
GGCGATCCAGCTGCCTCTTTTATGGNNNATTTAAGTCTGTTCCTACCCAAACTTGTGA
CAGGACAGAGCGCCTGTTCTCAGAAAGGGAAAAAGGCCTACCGACAATTAGAGAAT
CGAGGTATAAATGTCATCTCTCGAGAGTGGAAGGCATCTATGGCGATGAGAATGGTT
TATCGCACACTTGGGCACTGCTCTATCATACGTTTTAGCCTGATCTACCGTGAGCTTC
ATCAGCGGGCTGGCACTGGCACTGTTAAAACAATCATAGCAGAAGCTGTTGARCAAT
CACGATTCTCTGGGCTATTGATGGTAAAAAATGTTCTGACCTAAATAATTACAAACA
AGGAAGGAGACATC-
AAATACATCCACTTGCTCCAGATAAACAGACAAGAGGGGAGCTGGCTCGTTTTGCAT
ATTGTGTAGGGAATCTGCAGAAACAT-
GCATCGATGCTCCATTTGCTCGAGGTCTGGGGCTTCCCGGGGTGACCCAAACGGGAC
ATGGGCCATTCCCCCGTTTATCTGCCATTGCCCTTGGGGTCGCATCGGTCTACCAAAG
CACACTCGCAGGGGTTAATATTGACCCTCGGCATCAGTCCCTGAAGGAAGCAGCCCA
CCCAGCTGAACTAGGGCTACAGAGCATCCGCAATGCAAGGAAATTGCCTGAAGCGG
AGATCTCACCGTGGAAGAACGAGAGGGACTACTTCAGTTCMCTCGGAGAAGTGAAA
AAAATCAACCAAGATCAAAGACAGCAGGAAGAGAGGGCTNNNNNN

>Eptesicus_fuscus{HM545133}

CTGTC-
GGCAAACGAGCTGCCTCTTTACGGCATCTTTAAGTCTGTTCCTACCCAAACTTGTGA
CAGGAGGGAGCGCCTGTTCTCAGAAGGTAGAAGAGGCCTACCAACAATTAGAGGAT
CGAGGTATCCATGTAATCTCTCGAGACTGGATGGCACCTGTGGGGATGAGAATCGCT
TATCGCACGCTT-
GTCACTGCTTTACCAGACGCTCTAGCCTCATCCACCATGAGCTTCATCAACGGGCTGG
CACTGGCGCTGTTAAAACCGTCATAGCAGGAGCTGTTGAGCAACCACGATACTCTGG
GCTGTTGACGGTAAAAAATGTTCTGACCTATGTAATTACAAACGAGGAAGGAGACAT
CAAAATACATCCCCCTGCTCGAGATAAACGGACAAGAGAGGCGCTGGCTCGTTTGGA
ATACTGTGTAAGGAACCTGCAGAAGCATGGCATCTGTNNNNNNNNNNNNNNNNNNN
NNNNNNNNNNNNNNNNNNNNNNNNNNNNNNNNNNNNNNNNNNNNNNNNNNNNNN
NNNNNNNNNNNNNNNNNNNNNNNNNNNNNNNNNNNNNNNNNNNNNNNNNNNNNN
NNNNNNNNNNNNNNNNNNNNNNNNNNNNNNNNNNNNNNNNNNNNNNNNNNNNNN
NNNNNNNNNNNNNNNNNNNNNNNNNNNNNNNNNNNNNNNNNNNNNNNNNNNNNN
NNNNNNNNNNNNNNNNNNNNNNNNNNNNNNNNNNNNNNNNNNNNNNNNNNNNNN
NNNNNNNNNNNNNNNNNNNNNNNNNNNNNNNN
