# Supplementary material for: Evolutionary maintenance of filovirus-like genes in bat genomes
Source: BMC Evol Biol. 2011 Nov 17;11:336. doi: 10.1186/1471-2148-11-336 (PMC3229293; doi:10.1186/1471-2148-11-336)
Supplement: Additional file 6 — Table S2. Site-by-site selection results and statistics for the open reading frame alignment of VP35-like genes in Myotis using two methods. Results of different site-specific tests of positive selection (Bayesian Ka/Ks with model M8; REL analysis in HyPhy) for the filovirus VP35-like gene in Myotis bats. [file 1471-2148-11-336-S6.PDF]

Table S2

Results of different site-specific tests of positive selection (Bayesian Ka/Ks in Selecton with model M8; REL analysis in HyPhy) for the filovirus VP35-like gene in *Myotis* bats. Values are displayed beside the amino acid residues of sequence 1 of the alignment including gaps. Confidence Intervals with lower bounds (LB) > 1 are considered significant or reliable sites for positive selection in Selecton. Significant sites for positive selection are colored red for each analysis. E[dN-dS] = Posterior mean of the dN-dS difference; PP = posterior probability; and BF = Bayes Factor

	Selecton			REL (HyPhy)		
	AA	Ka/Ks	CI [LB,UB]	E[dN-dS]	PP	BF
1	M	0.32	[1.8e-11,1.5]	-0.69	0.14	0.23
2	S	0.55	[1.8e-11,1.5]	-0.30	0.21	0.38
3	L	0.52	[1.8e-11,1.5]	-0.21	0.26	0.50
4	E	0.41	[1.8e-11,1.5]	-0.35	0.21	0.38
5	Q	0.48	[1.8e-11,1.5]	-0.31	0.23	0.43
6	F	1.5	[1.5,1.5]	1.16	0.99	209.41
7	I	0.48	[1.8e-11,1.5]	-0.34	0.20	0.35
8	Q	1.5	[1.5,1.5]	1.14	0.99	174.40
9	Q	1.4	[0.23,1.5]	1.03	0.93	19.33
10	I	0.36	[1.8e-11,1.5]	-0.44	0.17	0.30
11	S	0.42	[1.8e-11,1.5]	-0.46	0.17	0.29
12	Q	1.4	[0.23,1.5]	1.02	0.93	17.90
13	L	0.53	[1.8e-11,1.5]	-0.51	0.23	0.42
14	T	1.5	[1.5,1.5]	1.12	0.99	169.03
15	D	0.35	[1.8e-11,1.5]	-0.41	0.19	0.35
16	H	1.5	[1.5,1.5]	1.19	1.00	354.82

17	C	0.52	[1.8e-11,1.5]	-0.44	0.18	0.32
18	D	0.35	[1.8e-11,1.5]	-0.41	0.19	0.35
19	R	0.46	[1.8e-11,1.5]	-0.35	0.20	0.36
20	I	1.4	[0.23,1.5]	1.04	0.92	16.43
21	K	1.5	[1.5,1.5]	1.13	0.99	159.30
22	E	0.39	[1.8e-11,1.5]	-0.36	0.21	0.38
23	A	1.5	[1.5,1.5]	1.20	1.00	471.21
24	M	0.32	[1.8e-11,1.5]	-0.69	0.14	0.23
25	T	0.42	[1.8e-11,1.5]	-0.30	0.21	0.39
26	S	0.46	[1.8e-11,1.5]	-0.32	0.21	0.38
27	L	0.44	[1.8e-11,1.5]	-0.72	0.22	0.41
28	T	1.5	[1.5,1.5]	1.19	1.00	374.65
29	S	0.5	[1.8e-11,1.5]	-0.42	0.17	0.29
30	C	0.59	[1.8e-11,1.5]	-0.39	0.18	0.32
31	M	0.32	[1.8e-11,1.5]	-0.69	0.14	0.23
32	E	0.39	[1.8e-11,1.5]	-0.36	0.21	0.38
33	K	0.42	[1.8e-11,1.5]	-1.23	0.19	0.33
34	Q	0.48	[1.8e-11,1.5]	-1.18	0.21	0.39
35	L	1.4	[0.23,1.5]	1.01	0.92	16.56
36	V	0.4	[1.8e-11,1.5]	-0.30	0.22	0.41
37	T	1.5	[1.5,1.5]	1.18	1.00	291.67
38	M	1.4	[0.23,1.5]	0.76	0.87	9.83
39	D	1.4	[0.23,1.5]	0.97	0.91	15.46
40	H	0.4	[1.8e-11,1.5]	-0.40	0.20	0.36
41	L	0.54	[1.8e-11,1.5]	-0.27	0.23	0.43
42	L	1.4	[0.23,1.5]	0.87	0.92	17.22
43	A	0.39	[1.8e-11,1.5]	-0.29	0.22	0.41

44	A	0.5	[1.8e-11,1.5]	-0.28	0.22	0.41
45	L	1.4	[0.23,1.5]	1.09	0.94	23.28
46	M	1.5	[1.5,1.5]	0.84	0.89	11.36
47	E	0.41	[1.8e-11,1.5]	-0.35	0.21	0.38
48	I	0.36	[1.8e-11,1.5]	-0.44	0.17	0.30
49	K	0.4	[1.8e-11,1.5]	-0.38	0.20	0.36
50	A	0.41	[1.8e-11,1.5]	-0.29	0.22	0.41
51	Q	0.65	[1.8e-11,1.5]	-0.19	0.35	0.78
52	L	0.68	[1.8e-11,1.5]	-0.12	0.35	0.78
53	P	0.65	[1.8e-11,1.5]	-0.14	0.35	0.78
54	D	0.35	[1.8e-11,1.5]	-0.41	0.19	0.35
55	Q	0.46	[1.8e-11,1.5]	-0.32	0.23	0.43
56	V	0.5	[1.8e-11,1.5]	-0.28	0.22	0.41
57	F	1.5	[1.5,1.5]	1.12	0.99	139.17
58	F	1.4	[0.23,1.5]	0.95	0.90	13.49
59	S	0.42	[1.8e-11,1.5]	-0.46	0.17	0.29
60	Q	0.48	[1.8e-11,1.5]	-0.31	0.23	0.43
61	S	0.42	[1.8e-11,1.5]	-0.46	0.17	0.29
62	L	0.44	[1.8e-11,1.5]	-0.28	0.23	0.43
63	L	0.52	[1.8e-11,1.5]	-0.21	0.26	0.50
64	S	0.46	[1.8e-11,1.5]	-0.32	0.21	0.38
65	M	1.5	[1.5,1.5]	0.42	0.93	18.38
66	S	0.46	[1.8e-11,1.5]	-0.32	0.21	0.38
67	S	0.46	[1.8e-11,1.5]	-0.32	0.21	0.38
68	K	1.4	[0.23,1.5]	1.01	0.92	16.22
69	V	1.4	[0.23,1.5]	1.06	0.93	19.42
70	N	0.37	[1.8e-11,1.5]	-0.43	0.19	0.33

71	Q	0.46	[1.8e-11,1.5]	-0.32	0.23	0.43
72	L	0.5	[1.8e-11,1.5]	-0.21	0.26	0.50
73	V	0.42	[1.8e-11,1.5]	-0.29	0.22	0.41
74	G	1.5	[1.5,1.5]	1.17	0.99	264.84
75	D	1.5	[1.5,1.5]	1.12	0.99	163.94
76	S	1.4	[0.23,1.5]	0.71	0.91	13.99
77	S	0.46	[1.8e-11,1.5]	-0.32	0.21	0.38
78	E	0.41	[1.8e-11,1.5]	-0.35	0.21	0.38
79	L	0.44	[1.8e-11,1.5]	-0.28	0.23	0.43
80	L	0.56	[1.8e-11,1.5]	-0.22	0.25	0.49
81	A	0.5	[1.8e-11,1.5]	-0.28	0.22	0.41
82	K	0.42	[1.8e-11,1.5]	-0.37	0.20	0.36
83	L	0.44	[1.8e-11,1.5]	-0.28	0.23	0.43
84	N	1.4	[0.23,1.5]	0.95	0.91	13.71
85	Y	1.4	[0.23,1.5]	0.99	0.92	17.43
86	L	0.56	[1.8e-11,1.5]	-0.24	0.24	0.46
87	P	1.4	[0.23,1.5]	1.07	0.93	20.32
88	V	0.42	[1.8e-11,1.5]	-0.29	0.22	0.41
89	M	0.32	[1.8e-11,1.5]	-0.69	0.14	0.23
90	S	1.4	[0.23,1.5]	1.08	0.94	21.97
91	G	1.4	[0.23,1.5]	1.06	0.93	19.42
92	P	0.44	[1.8e-11,1.5]	-0.28	0.23	0.43
93	A	1.4	[0.23,1.5]	0.73	0.91	13.84
94	T	0.45	[1.8e-11,1.5]	-0.31	0.21	0.39
95	S	0.55	[1.8e-11,1.5]	-0.30	0.21	0.38
96	T	0.4	[1.8e-11,1.5]	-0.31	0.21	0.39
97	L	0.54	[1.8e-11,1.5]	-10.05	0.03	0.04

98	E	1.4	[0.23,1.5]	1.02	0.92	17.65
99	A	0.41	[1.8e-11,1.5]	-0.29	0.22	0.41
100	A	0.39	[1.8e-11,1.5]	-0.29	0.22	0.41
101	G	0.46	[1.8e-11,1.5]	-0.29	0.22	0.41
102	A	0.41	[1.8e-11,1.5]	-0.29	0.22	0.41
103	N	1.4	[0.23,1.5]	1.01	0.92	15.57
104	T	1.5	[1.5,1.5]	0.86	0.97	43.33
105	Q	0.48	[1.8e-11,1.5]	-0.31	0.23	0.43
106	E	1.4	[0.23,1.5]	1.02	0.92	17.26
107	H	0.4	[1.8e-11,1.5]	-1.68	0.18	0.32
108	R	1.4	[0.23,1.5]	1.03	0.92	16.79
109	R	0.47	[1.8e-11,1.5]	-0.36	0.19	0.34
110	P	0.44	[1.8e-11,1.5]	-0.71	0.22	0.41
111	P	0.54	[1.8e-11,1.5]	-0.26	0.23	0.43
112	P	0.46	[1.8e-11,1.5]	-0.27	0.23	0.43
113	G	0.48	[1.8e-11,1.5]	-0.30	0.21	0.39
114	P	0.54	[1.8e-11,1.5]	-0.26	0.23	0.43
115	I	1.4	[0.23,1.5]	1.04	0.92	16.59
116	L	0.5	[1.8e-11,1.5]	-0.21	0.26	0.50
117	A	0.41	[1.8e-11,1.5]	-0.29	0.22	0.41
118	T	1.5	[1.5,1.5]	1.20	1.00	445.37
119	L	1.4	[0.23,1.5]	1.10	0.94	23.78
120	E	0.41	[1.8e-11,1.5]	-1.23	0.19	0.34
121	R	0.54	[1.8e-11,1.5]	-0.18	0.27	0.53
122	H	1.5	[1.5,1.5]	0.31	0.92	16.17
123	G	0.46	[1.8e-11,1.5]	-0.29	0.22	0.41
124	A	1.4	[0.23,1.5]	0.71	0.90	13.25

125	R	0.46	[1.8e-11,1.5]	-0.35	0.20	0.36
126	P	1.5	[1.5,1.5]	0.96	0.98	59.71
127	T	0.42	[1.8e-11,1.5]	-0.30	0.21	0.39
128	D	0.35	[1.8e-11,1.5]	-0.41	0.19	0.35
129	T	1.4	[0.23,1.5]	0.61	0.89	11.80
130	L	0.54	[1.8e-11,1.5]	-0.27	0.23	0.43
131	T	0.4	[1.8e-11,1.5]	-0.31	0.21	0.39
132	S	0.46	[1.8e-11,1.5]	-0.32	0.21	0.38
133	D	0.35	[1.8e-11,1.5]	-0.41	0.19	0.35
134	I	0.38	[1.8e-11,1.5]	-0.37	0.20	0.35
135	P	0.47	[1.8e-11,1.5]	-3.98	0.15	0.26
136	G	0.48	[1.8e-11,1.5]	-0.30	0.21	0.39
137	Y	1.5	[1.5,1.5]	-9.68	0.02	0.03
138	A	1.4	[0.23,1.5]	1.07	0.93	19.32
139	E	1.5	[1.5,1.5]	1.15	0.99	187.96
140	A	1.4	[0.23,1.5]	1.06	0.93	19.31
141	A	0.4	[1.8e-11,1.5]	-0.75	0.21	0.39
142	E	0.41	[1.8e-11,1.5]	-0.35	0.21	0.38
143	A	0.5	[1.8e-11,1.5]	-0.28	0.22	0.41
144	E	0.41	[1.8e-11,1.5]	-0.35	0.21	0.38
145	K	0.42	[1.8e-11,1.5]	-0.37	0.20	0.36
146	K	0.4	[1.8e-11,1.5]	-0.38	0.20	0.36
147	V	1.4	[0.23,1.5]	0.95	0.90	13.24
148	-	1.4	[0.23,1.5]	-0.52	0.80	5.73
149	-	0.44	[1.8e-11,1.5]	-0.28	0.23	0.43
150	-	0.45	[1.8e-11,1.5]	-0.26	0.24	0.45
151	-	1.5	[1.5,1.5]	1.15	0.99	200.58

152	-	1.4	[0.23,1.5]	1.06	0.94	20.85
153	-	0.53	[1.8e-11,1.5]	-0.26	0.24	0.45
154	-	1.5	[1.5,1.5]	1.17	1.00	296.82
155	-	0.5	[1.8e-11,1.5]	-0.28	0.23	0.43
156	-	0.44	[1.8e-11,1.5]	-0.33	0.23	0.42
157	-	1.4	[0.23,1.5]	0.99	0.92	16.24
158	-	0.53	[1.8e-11,1.5]	-0.61	0.23	0.43
159	-	0.52	[1.8e-11,1.5]	-0.76	0.22	0.40
160	-	1.4	[0.23,1.5]	1.09	0.94	23.46
161	L	0.52	[1.8e-11,1.5]	-0.21	0.26	0.50
162	P	0.44	[1.8e-11,1.5]	-0.28	0.23	0.43
163	L	0.5	[1.8e-11,1.5]	-0.21	0.26	0.50
164	V	1.5	[1.5,1.5]	0.74	0.96	31.89
165	P	0.54	[1.8e-11,1.5]	-0.26	0.23	0.43
166	T	0.51	[1.8e-11,1.5]	-0.30	0.21	0.39
167	E	0.39	[1.8e-11,1.5]	-0.36	0.21	0.38
168	F	0.52	[1.8e-11,1.5]	-0.41	0.17	0.30
169	V	0.42	[1.8e-11,1.5]	-0.65	0.21	0.39
170	R	1.4	[0.23,1.5]	1.10	0.95	24.99
171	V	0.5	[1.8e-11,1.5]	-0.28	0.22	0.41
172	L	0.54	[1.8e-11,1.5]	-0.27	0.23	0.43
173	T	0.42	[1.8e-11,1.5]	-0.30	0.21	0.39
174	S	1.4	[0.23,1.5]	0.95	0.90	13.12
175	H	1.4	[0.23,1.5]	-0.30	0.81	6.32
176	L	0.52	[1.8e-11,1.5]	-0.35	0.25	0.49
177	T	1.5	[1.5,1.5]	0.77	0.96	34.20
178	G	0.46	[1.8e-11,1.5]	-0.29	0.22	0.41

179	P	0.52	[1.8e-11,1.5]	-10.29	0.02	0.03
180	R	1.4	[0.23,1.5]	1.07	0.93	19.32
181	T	0.42	[1.8e-11,1.5]	-4.93	0.13	0.21
182	A	1.4	[0.23,1.5]	1.06	0.93	19.25
183	F	0.43	[1.8e-11,1.5]	-0.45	0.17	0.30
184	H	0.4	[1.8e-11,1.5]	-1.74	0.18	0.31
185	E	0.39	[1.8e-11,1.5]	-0.36	0.21	0.38
186	L	0.56	[1.8e-11,1.5]	-0.22	0.25	0.49
187	V	0.43	[1.8e-11,1.5]	-0.68	0.21	0.39
188	S	0.52	[1.8e-11,1.5]	-0.57	0.23	0.42
189	A	0.45	[1.8e-11,1.5]	-0.65	0.22	0.39
190	I	1.4	[0.23,1.5]	1.04	0.92	16.58
191	A	0.39	[1.8e-11,1.5]	-0.29	0.22	0.41
192	V	1.5	[1.5,1.5]	1.19	1.00	676.63
193	V	0.44	[1.8e-11,1.5]	-0.29	0.22	0.41
194	S	0.5	[1.8e-11,1.5]	-0.42	0.17	0.29
195	R	0.54	[1.8e-11,1.5]	-0.18	0.27	0.53
196	D	0.44	[1.8e-11,1.5]	-0.37	0.20	0.35
197	S	0.46	[1.8e-11,1.5]	-0.32	0.21	0.38
198	H	0.41	[1.8e-11,1.5]	-1.85	0.18	0.31
199	D	0.35	[1.8e-11,1.5]	-0.41	0.19	0.35
200	L	0.5	[1.8e-11,1.5]	-0.21	0.26	0.50
201	Q	0.48	[1.8e-11,1.5]	-0.31	0.23	0.43
202	V	0.42	[1.8e-11,1.5]	-0.29	0.22	0.41
203	A	0.49	[1.8e-11,1.5]	-0.53	0.22	0.40
204	M	0.32	[1.8e-11,1.5]	-0.69	0.14	0.23
205	D	0.44	[1.8e-11,1.5]	-0.37	0.20	0.35

206	H	1.4	[0.23,1.5]	0.98	0.92	16.56
207	F	0.52	[1.8e-11,1.5]	-0.41	0.17	0.30
208	N	0.37	[1.8e-11,1.5]	-0.43	0.19	0.33
209	R	0.54	[1.8e-11,1.5]	-0.37	0.27	0.53
210	E	0.41	[1.8e-11,1.5]	-0.35	0.21	0.38
211	L	0.5	[1.8e-11,1.5]	-0.21	0.26	0.50
212	M	1.5	[1.5,1.5]	1.18	1.00	505.14
213	D	0.39	[1.8e-11,1.5]	-1.39	0.18	0.32
214	G	0.43	[1.8e-11,1.5]	-0.31	0.21	0.39
215	S	1.4	[0.23,1.5]	1.03	0.92	15.61
216	S	0.51	[1.8e-11,1.5]	-0.25	0.24	0.46
217	A	0.39	[1.8e-11,1.5]	-0.29	0.22	0.41
218	H	0.4	[1.8e-11,1.5]	-0.40	0.20	0.36
219	A	0.39	[1.8e-11,1.5]	-0.29	0.22	0.41
220	A	0.5	[1.8e-11,1.5]	-0.28	0.22	0.41
221	I	0.36	[1.8e-11,1.5]	-0.44	0.17	0.30
222	I	0.36	[1.8e-11,1.5]	-0.44	0.17	0.30
223	S	0.55	[1.8e-11,1.5]	-0.30	0.21	0.38
224	I	0.48	[1.8e-11,1.5]	-0.34	0.20	0.35
225	T	0.4	[1.8e-11,1.5]	-0.31	0.21	0.39
226	R	1.5	[1.5,1.5]	1.20	1.00	536.62
227	R	0.47	[1.8e-11,1.5]	-0.83	0.19	0.34
228	C	0.52	[1.8e-11,1.5]	-0.44	0.18	0.32
229	E	0.41	[1.8e-11,1.5]	-0.35	0.21	0.38
230	H	1.5	[1.5,1.5]	1.11	0.99	136.99
231	F	0.43	[1.8e-11,1.5]	-0.45	0.17	0.30
232	R	0.53	[1.8e-11,1.5]	-0.24	0.24	0.46

233	N	0.46	[1.8e-11,1.5]	-0.38	0.19	0.33
234	C	0.59	[1.8e-11,1.5]	-0.39	0.18	0.32
235	E	0.39	[1.8e-11,1.5]	-0.36	0.21	0.38
236	A	0.39	[1.8e-11,1.5]	-0.29	0.22	0.41
237	P	0.48	[1.8e-11,1.5]	-0.27	0.23	0.43
238	T	0.42	[1.8e-11,1.5]	-0.30	0.21	0.39
239	M	1.5	[1.5,1.5]	1.13	0.99	199.56
240	Q	0.48	[1.8e-11,1.5]	-0.31	0.23	0.43
241	V	0.42	[1.8e-11,1.5]	-0.29	0.22	0.41
242	T	0.4	[1.8e-11,1.5]	-0.31	0.21	0.39
243	S	0.52	[1.8e-11,1.5]	-0.27	0.23	0.43
244	K	0.42	[1.8e-11,1.5]	-0.37	0.20	0.36
245	S	0.5	[1.8e-11,1.5]	-0.42	0.17	0.29
246	Q	0.48	[1.8e-11,1.5]	-0.31	0.23	0.43
247	I	0.38	[1.8e-11,1.5]	-0.37	0.20	0.35
248	P	0.46	[1.8e-11,1.5]	-0.27	0.23	0.43
249	K	1.5	[1.5,1.5]	1.14	0.99	225.88
250	A	0.41	[1.8e-11,1.5]	-0.29	0.22	0.41
251	C	1.5	[1.5,1.5]	1.11	0.99	138.21
252	H	0.4	[1.8e-11,1.5]	-0.40	0.20	0.36
253	G	0.53	[1.8e-11,1.5]	-0.30	0.21	0.39
254	R	0.46	[1.8e-11,1.5]	-0.35	0.20	0.36
255	L	0.44	[1.8e-11,1.5]	-0.28	0.23	0.43
256	R	0.47	[1.8e-11,1.5]	-0.36	0.19	0.34
257	D	0.35	[1.8e-11,1.5]	-0.41	0.19	0.35
258	V	0.42	[1.8e-11,1.5]	-0.29	0.22	0.41
259	P	0.49	[1.8e-11,1.5]	-0.60	0.22	0.42

260	E	0.42	[1.8e-11,1.5]	-0.34	0.21	0.39
261	G	0.44	[1.8e-11,1.5]	-0.30	0.22	0.40
262	A	1.4	[0.23,1.5]	1.08	0.94	21.24
263	K	0.41	[1.8e-11,1.5]	-0.37	0.21	0.37
264	T	1.5	[1.5,1.5]	1.20	1.00	473.76
265	L	0.51	[1.8e-11,1.5]	-0.20	0.26	0.51
266	G	0.47	[1.8e-11,1.5]	-0.28	0.23	0.43
267	R	1.5	[1.5,1.5]	1.19	1.00	456.29
268	G	0.47	[1.8e-11,1.5]	-0.68	0.22	0.41
269	W	0.59	[1.8e-11,1.5]	-0.54	0.22	0.39
270	V	1.4	[0.23,1.5]	1.06	0.93	19.82
271	Y	0.48	[1.8e-11,1.5]	-0.36	0.23	0.42
272	I	0.37	[1.8e-11,1.5]	-0.43	0.18	0.32
273	Y	0.48	[1.8e-11,1.5]	-0.36	0.23	0.42

==
