# Supplementary material for: Evolutionary maintenance of filovirus-like genes in bat genomes
Source: BMC Evol Biol. 2011 Nov 17;11:336. doi: 10.1186/1471-2148-11-336 (PMC3229293; doi:10.1186/1471-2148-11-336)
Supplement: Additional file 7 — Figure S5. RT-PCR of VP35-like region from Myotis lucifugus. RT-PCR of the filovirus VP35-like region and actin controls in Myotis lucifugus from two replicates each from two tissues. [file 1471-2148-11-336-S7.PDF]

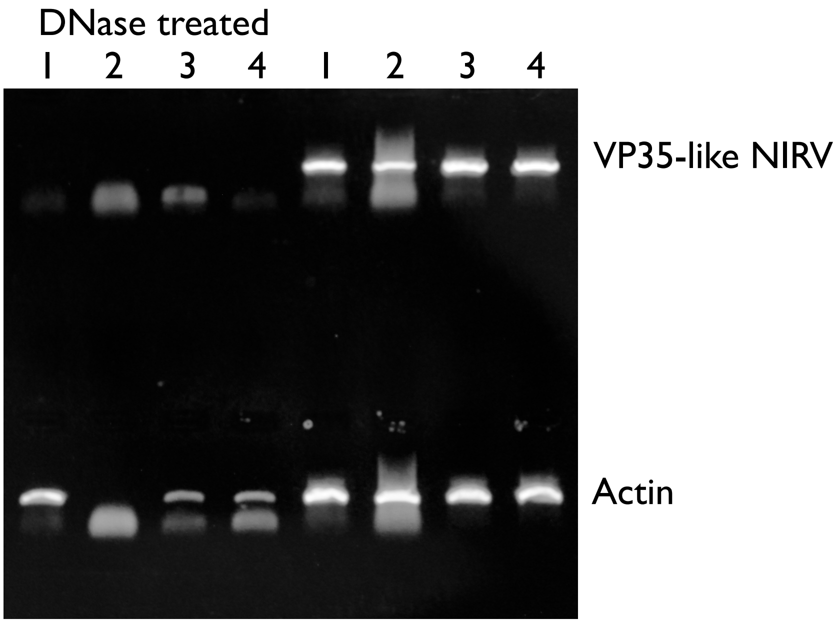

Figure S5. RT-PCR of the filovirus VP35-like region in *Myotis lucifugus* from two replicates each from two tissues (1-2 lung; 3-4 liver). The top row used VP35like NIRV primers while the bottom row used actin primers as a positive control for detecting RNA expression. The first four columns are treated with DNase I to remove DNA as a template. The last four columns are positive controls for PCR and size standards. There was no detectable RT-PCR band from the RNA extractions using the VP35-like NIRV primers. Three of four assays for the actin primers yielded RT-PCR product of the expected size from the RNA extractions.
