# Supplementary material for: Evolutionary maintenance of filovirus-like genes in bat genomes
Source: BMC Evol Biol. 2011 Nov 17;11:336. doi: 10.1186/1471-2148-11-336 (PMC3229293; doi:10.1186/1471-2148-11-336)
Supplement: Additional file 8 — Table S3. Table showing the specimen details for bats assayed for filovirus-like sequences. Origin of specimens used for the attempted isolation of filovirus-like sequences in the present study. [file 1471-2148-11-336-S8.PDF]

Table S3. Origin of specimens used for the attempted isolation of filovirus-like sequences in the present study.

Collectors: E.A. Rickart, J.L. Sedlock, L.R. Heaney, B.D. Patterson, L. Davalos, P. Myers, E. Suazo; Museums: Field Museum of Natural History, Texas Tech University Museum, American Museum of Natural History

Species	Family	Voucher number	State/Province	Country
Myotis albescens	Vespertilionidae	MUSM 15240	Loreto	Peru
Myotis annectans	Vespertilionidae	AMNH 272378	Ha Tinh	Vietnam
Myotis blythii	Vespertilionidae	FMNH 140372	Malakand	Pakistan
Myotis lucifugus	Vespertilionidae	FMNH 172384	Minnesota	USA
Myotis lucifugus	Vespertilionidae	Bat # 5866-10 NYS Health Dept.	(Tompkins Co.) New York	USA
Myotis muricola browni	Vespertilionidae	FMNH 167239	Kalinga	Philippine Is
Myotis nigricans	Vespertilionidae	FMNH 162544	Tarija	Bolivia
Myotis oxyotus	Vespertilionidae	FMNH 174938	Cusco	Peru
Myotis riparius	Vespertilionidae	MUSM 13224	Loreto	Peru
Myotis septentrionalis	Vespertilionidae	Bat # 10-05851 NYS Health Dept.	(Westchester Co.) New York	USA
Myotis horsfieldii jeannei	Vespertilionidae	FMNH 177466	Luzon	Philippine Is
Eptesicus fuscus	Vespertilionidae	ALR130	M Trinidad Sanchez	Dominican Republic
Miniopterus australis paululus	Vespertilionidae	FMNH 175414	Luzon	Philippine Is
Pipistrellus javanicus meyeni	Vespertilionidae	FMNH 167237	Luzon	Phillipine Is
Scotophilus kuhlii	Vespertilionidae	LRH 8051	Luzon	Philippine Is
Lasiurus borealis borealis	Vespertilionidae	FMNH 175342	Illinois	USA
Pteronotus quadridens	Mormoopidae	ALR5	Arecibo	Puerto Rico
Pteronotus parnelli	Mormoopidae	ALR38	Toa Alta	Puerto Rico
Mormoops blainvillii	Mormoopidae	ALR57	Aguadilla	Puerto Rico
Brachyphylla cavernarum	Phyllostomidae	ALR69	Arecibo	Puerto Rico
Phyllonycteris lipoeyi	Phyllostomidae	ALR161	Sanchez Ramirez	Dominican Republic
Diphylla ecaudata	Phyllostomidae	TK34981	Colon	Honduras
Noctilio leporinus	Noctilionidae	ALR160	Sanchez Ramirez	Dominican Republic
